# Does engagement with frontline health workers improve maternal and child healthcare utilisation and outcomes in India?

**DOI:** 10.1186/s12960-021-00592-1

**Published:** 2021-04-01

**Authors:** Anu Rammohan, Srinivas Goli, Shashi Kala Saroj, C. P. Abdul Jaleel

**Affiliations:** 1 The University of Western Australia (M251), Crawley, WA Australia; 2 Jawaharlal Nehru University, New Delhi, India; 3 UNICEF National Centre of Excellence and Advanced Research on Diets, Lady Irwin College, University of Delhi, New Delhi, India; 4 Present Address: Australia India Institute, UWA Public Policy Institute, University of Western Australia, Crawley, WA Australia

**Keywords:** Frontline health workers, Health services, Maternal health, Child health, Child mortality, Antenatal care, Delivery care, Postnatal care, India

## Abstract

**Background:**

Poor Maternal and Child Health (MCH) outcomes pose challenges to India’s ability to attain Goal-3 of the Sustainable Development Goals (SDGs). The government of India strengthened the existing network of frontline health workers (FHWs), under its National Rural Health Mission in 2005 and subsequent National Urban Health Mission in 2013 as a strategy to mitigate the shortage of skilled health workers and to provide affordable healthcare services. However, there is a lack of robust national-level empirical analysis on the role of maternal engagement with FHWs in influencing the level of maternal and child health care utilisation and child health outcomes in India.

**Methods:**

Using data from the nationally representative Indian National Family Health Survey (NFHS) 2015–2016, this paper aims to investigate the intensity of engagement of FHWs with married women of child-bearing age (15–49 years), its influence on utilisation of maternal and child healthcare services, and child health outcomes. Our empirical analyses use multivariate regression analyses, focusing on five maternal and child health indicators: antenatal care visits (ANC) (4 or > 4 times), institutional delivery, full-immunisation of children, postnatal care (PNC) (within 2 days of delivery), and child survival.

**Results:**

Our analysis finds that maternal engagement with FHWs is statistically significant and a positive predictor of maternal and child health care utilisation, and child survival. Further, the level of engagement with FHWs is particularly important for women from economically poor households. Our robustness checks across sub-samples of women who delivered only in public health institutions and those from rural areas provides an additional confidence in our main results.

**Conclusions:**

From a policy perspective, our findings highlight that strengthening the network of FHWs in the areas where they are in shortage which can help in further improving the utilisation of maternal and child healthcare services, and health outcomes. Also, the role of FHWs in the government health system needs to be enhanced by improving skills, working environment, and greater financial incentives.

**Supplementary Information:**

The online version contains supplementary material available at 10.1186/s12960-021-00592-1.

## Background

Despite a decline in Under-Five Mortality Rate (U5MR) in India from 126 per 1000 live births in 1990 to 37 per 1000 live births in 2018, poor Maternal and Child Health (MCH) outcomes pose challenges to India’s ability to attain Goal-3 of the Sustainable Development Goals (SDGs). Specifically, Goal 3.2 seeks to ‘reduce the global neonatal mortality and U5MR to at least 12 per 1000 live births and 25 per 1000 live births by 2030 [1, 2]. Evidence from India’s countrywide representative 2015–2016 National Family Health Survey (NFHS) show that 21% of live births did not have skilled attendance, 49% of pregnant women did not receive basic antenatal care, and approximately 76% of new-born babies did not receive health care within 2 days of birth. Furthermore, the proportion of children who did not receive any vaccination has increased in 2015–2016 compared to 2005–2006 [3].

Researchers have attributed these poor maternal and child health outcomes to low levels of health care utilisation during pregnancy and childbirth [4–6]. Low levels of health care utilisation may be due to demand-side factors such as poverty, lack of education, rural residence or other socio-economic deprivations [7–11]. Other researchers have proposed supply-side reasons such as poor availability, accessibility, and inequality in accessing health care resources [12–16].

Studies from a wide range of settings have shown that greater access to Frontline Health Workers (FHWs) or Community Health Workers (CHW) can greatly improve maternal and child health care utilisation and outcomes. FHWs play an important role in disseminating community-based health information and encouraging the utilisation of MCH and family planning services [13, 17–23]. FHWs directly interact with women and their family members, particularly in poor and remote disadvantaged communities [21, 23–25]. They perform tasks such as increasing awareness of hygiene and sanitation practices, making a timely referral of patients to health facilities, monitoring, and play an important role in the supervision of health and nutrition programmes at the community level, reporting of vital events and motivating women to access essential care from health facilities [26, 27]. Health workers, in particular, FHWs are an integral part of improving MCH care utilisation, particularly in rural areas of developing countries, with low access to health services [19]. FHWs are typically health care workers who deliver routine and essential services in public health or medical practice. They encompass a wide variety of local healthcare providers ranging from salaried-staff, midwives, nurses to home-based caregivers, and volunteers, with their functions varying across countries [28, 29].

In India, although the role of FHWs is not empirically analysed using individual-level information at the national level, macro-levels (correlation between MCH care indicators and recruitment of community health workers) [13] and studies using randomised [14] and provincial level case studies [18, 30–32] have shown that greater access to FHWs can greatly improve MCH care utilisation and outcomes,. In India’s health care delivery system, FHWs have an integral role to play in the supply of MCH services, particularly disseminating information about the government’s flagship health care programmes among eligible beneficiaries such as pregnant women, mothers and their children [33]. Previous research suggests that the utilisation of maternal and child healthcare (MCH) services has increased substantially since the launch of flagship MCH programmes (i.e. Integrated Child Development Schemes [ICDS] and National Health Mission (NHM) [4, 34–36].

Furthermore, although workers such as Auxiliary Nurse Midwives (ANM) and Anganwadi workers have been around since the 1950s and 1970s, respectively, the aim of enhancing primary health care in villages as a link between health care services and the community has received prominence since 2005 [37, 38]. The existing network of FHWs was strengthened in 2005, under the Government of India’s National Rural Health Mission, and in 2013 under the National Urban Health Mission, as a strategy to mitigate the shortage of skilled health workers, and to provide affordable healthcare services to vulnerable groups [13, 21, 37–40]. The FHWs perform multiple roles that include identifying, motivating, tracking and facilitating basic maternal and child healthcare and nutrition services to pregnant women and children under the age of five often in team (for the detailed description see Box 1). Therefore, it is important to identify the role of FHWs in improving access to MCH care. However, there is a lack of robust national-level evidence on the association of engagement with FHWs and MCH care and outcomes.

**Table:** Box 1. Roles of frontline health workers in the public health system in India. Source: [37, 38]

Within the Ministry of Health and Family Welfare (MoHFW), Primary Health Centres (PHCs) are the first contact between community and a doctor in rural areas in India. PHCs provide basic preventive, promotive and curative, and outreach services through Sub-centres (SC), Community Health Centre (CHCs), Rural Family Welfare Centers. A SC covers a population of 3000–3500 and each SC is proposed to have one or two Auxiliary Nurse Midwives (ANMs) (minimum education: 10, +2 passed and have 18 months training from the Indian Nursing Council (INC)). Lady Health Visitor (LHV) (promoted ANM after 5 years experience) and Multi-Purpose Health Worker (MPHW) are to support the supervision and technical guidance to the ANMs in sub-centres MoHFW also introduced Accredited Social Health Activists (ASHAs) in 2005. They are incentive-based female community volunteers who should be literate and married local resident of the community (minimum education: 8th passed and have 23 days training) serve as an interface between the community and public health system at a village level of population 1000 (500 in tribal/hilly areas), similarly, in the slum population of urban areas (2500 population). Below briefly described the key roles of AWW, ASHA and ANMs Anganwadi worker (AWW): Anganwadi workers form a critical part of the Integrated Child Development Services (ICDS) programme, whose mandate is to provide pre-school education for children under six, and nutritional support and healthcare for children and pregnant or lactating mothers, to reduce mortality, morbidity, and malnutrition. AWWs run Anganwadi centres (AWCs)—the village- or slum-level delivery mechanism of the ICDS—and work with Accredited Social Health Activists (ASHAs) and Auxiliary Nurse Midwives (ANMs) to offer a package of six services. They fall within the purview of the MoWCD Accredited Social Health Activist (ASHA): ASHAs are part-time, trained community health volunteers, who work as an interface between the community and the public health system. They fall within the ambit of the MoHFW, and are a key component of the National Rural Health Mission (now called the National Health Mission). They are selected from the village to which they are accountable, and their role entails tracking pregnant women and newborns, delivering key health-related information, and promoting the better health-seeking behaviour, whether it’s in the home or at a medical institution Auxiliary Nurse Midwife (ANM): ANMs work at health sub-centres, who provide healthcare services at the village-level and are the closest service provider to the community, within the health system. ANMs have preliminary qualifications in midwifery and maternal and child health. They provide a range of services, such as dispensing medication, immunisation and family planning, assisting with deliveries, etc. Multi-Purpose Health Worker (MPHW): Multi-Purpose Health Worker (MPHW) are to support the supervision and technical guidance to the ANMs in sub-centres	

A PubMed search for studies on maternal and child health care with keywords of “determinants of maternal and child health care in India” shows that just two out of the top 100 search results (studies) have investigated the role of FHWs/CHWs in MCH care and outcomes. Thus, our study seeks to address the research gap on the role of maternal engagement with FHWs in influencing the level of MCH care utilisation and child health outcomes in India.

Against this background, our paper uses the nationally representative unit level information from Indian NFHS to empirically analyse the level of engagement between the FHWs and married women of child-bearing age (15–49 years), and its influence on the utilisation of MCH services and child health and survival outcomes. Our empirical analyses focus on five maternal and child health indicators—antenatal care visits (ANC) (4 or > 4 times), institutional delivery, full-immunisation of children, postnatal care (PNC) (within 2 days of delivery), and child survival.

Our paper makes several contributions to the literature. Firstly, previous research from India has focused mainly on the quality of service delivery system by FHWs [39–41], their impact on health inequality [31, 42, 43], maternal and new-born health [14, 30], and maternity care [13, 18]. However, a review conducted by [44] suggests that these studies use data that are based on macro-level (district level) analyses [14], provincial level [18, 30–32], experimental design [14] or case studies, and reviews studies [40], which makes it difficult to generalise their findings to the national level. Although, Agrawal and colleagues [41] evaluated the impact of introduction of Accredited Social Health Activist (ASHA) programme on utilisation of maternity services at the all India level, our study reports the effect of direct one-to-one interaction effect of FHWs as team on MCH care and outcome. Providing national-level evidence on the influence of direct interaction of women with FHWs on MCH care and outcomes is particularly critical from a policy perspective, given the heterogeneity in propensities of health care utilisation across various geographical regions, socio-economic, and demographic groups.

Secondly, considering the federal structure of India’s health care system and the diverse range of health policies and programmes across different states [45], it is important to identify the multi-dimensional roles of FHWs as team in enhancing MCH care and outcomes at the national level. In this direction, we use a comprehensive definition of FHWs encompassing various nomenclatures, work profiles, or functions used at the operational level in the government flagships programmes in different states. We have used 48 questions about the interactions of women with all categories of FHWs from multiple sections of the questionnaire schedule which were repeatedly asked at the different stages of pregnancy, childbirth, and new-born care in different forms, which serve as strong robustness checks for response errors. Thus, our measure of FHWs provides a robust tool to capture their influence in enhancing MCH care and outcomes.

Thirdly, we contribute to the literature by investigating if engagement with FHWs affects MCH utilisation differentially across different sub-sample of populations: who delivered in public health hospitals and residing in rural areas. In other words, are women delivered in public health hospitals or those who belong to rural areas are more likely to benefit from engaging with FHWs than their counterparts? Since the flagship health programmes largely target vulnerable rural women by strengthening the public health care system, thus the robustness of our results were tested using sub-sample analyses [46]. Moreover, relative to previous studies, we use a more comprehensive measure of the level of FHWs engagement with women at different stages of pregnancy, delivery and postnatal care. Thus, our study significantly adds to filling the research gap with national-level evidence.

## Methods

### Data and sampling

The data for this study come from the fourth round of India’s 2015–2016 National Family Health Survey (NFHS-4). The NFHS-4 is a nationally representative dataset of Indian households conducted by the International Institute for Population Sciences (IIPS), under the stewardship of the Ministry of Health and Family Welfare (MoHFW), Government of India. The household survey covered a sample of 601,509 households and 699,686 women aged 15–49 years. The survey used multi-stage cluster sampling with an overall response rate of 98%. Details of sample design, including sampling frame and survey implementation, are provided elsewhere [3]. Moreover, the data used for this study are publicly available at The Demographic Health Surveys [DHS] website, thus do not require ethical approval [47].

The data on MCH care, outcomes, and female’s engagement with health workers come from the Women’s questionnaire, which was administered to women aged 15–49 years. Given the focus of our analysis on MCH care and outcomes, we only include those women who gave birth in the 5 years prior to the survey. The Women’s questionnaire contains detailed information on the birth histories and details of all children born in the last 5 years. For these mothers, the survey provides detailed information on the socio-economic and demographic characteristics of the respondent and their households. Our final sample consists of 259,627 women aged 15–49 years (with 130,132 from poor households and 129,495 from non-poor households).

### Estimation strategy

#### Outcome variables

The outcome variables in this study include five key MCH indicators. Four of these indicators relate to MCH services utilisation, while we also include child survival as an additional outcome indicator. Specifically, we include the following outcome variables: (i) *ANC visits* = 1 if the respondent made at least 4+ antenatal visits during her last pregnancy, 0 otherwise; (ii) *Institutional delivery* = 1 if the birth was at a public/private institution, 0 otherwise; (iii) *Children’s Full Immunisation (CFI)* = 1 if a child (aged 12–23 months) received the full prescribed set of vaccinations. These are *BCG (one dose), DPT (three doses), Polio (three doses), and Measles (one dose)*, 0 otherwise; (iv) *Postnatal care (PNC)*—whether the child received PNC from a doctor or skilled medical personnel in the first 2 days of birth; and finally, (v) *Under-five child survival—*whether or not the child is alive.

#### Explanatory variables

Our main explanatory variable is an index called the *Frontline Health Worker Engagement Index (FHWEI),* which is constructed using information from responses to 48 questions on the female respondent’s engagement with the following categories of FHWs: *ANM/ASHA/Midwife/Lady health worker or other health workers/Multipurpose worker [MPW]/Anganwadi worker.* Following [48] method of index construction using non-linear variables, the 48 variables considered for FHWEI have been dichotomised into two categories: ‘engagement = 1’ and ‘no engagement = 0’. The reliability of the variables used in the construction of the index is measured using Cronbach’s coefficient. An alpha value of 0.94 indicates a high scale of reliability of the FHWEI (Table 4). The composite score of FHWEI was divided into three equal categories, i.e. ‘Low’, ‘Medium’, and ‘High’ quality of engagement.

Additionally, we include several control variables relating to the female respondent and her household’s socio-economic and demographic characteristics. These include the child’s birth order, mother’s age (categorised into seven discrete age groups), education level (illiterate, primary, secondary, higher, don’t know), and occupation (not working/white collar/agricultural activities/service or manual worker/don’t know or missing). Similarly, we include partner’s educational level (illiterate, primary, secondary, higher, don’t know), partner’s occupation (not working/white collar/agricultural activities/service or manual worker/don’t know or missing). Other control variables include the respondent’s religion (Hindu, Muslim, Christians, Others), *Caste* (Others, Scheduled *Castes* [SCs], Scheduled Tribes [STs], Other Backward Classes [OBCs], and Not reported/ Missing), place of residence (rural/urban) and geographical region (North, Central, Eastern, North Eastern, Western and Southern).

#### Empirical models

We have used two sets of empirical models: (1) binary logistic regression (BLR) models; (2) Cox proportional hazard models along with Kaplan–Meier plots for showing child survival estimates by FHWEI.

##### Binary logistic regression

Given that four of our outcome variables are binary in nature, we have estimated BLR models for each of four MCH care variables to demonstrate the influence of engagement of FHWE on MCH utilisation measures. The advantage of logistic regression analysis is that it requires no assumption about the distribution of the independent variables, and the regression coefficient can be interpreted in terms of odds ratios. For each of the outcome variables, in addition to the full sample, we have estimated BLR models for two sub-sets of samples: ‘poor’ women and ‘non-poor’ women. The same procedure was also followed in robustness checks conducted on samples of ‘women delivered in public sector health facilities only’ and for women from ‘rural areas’. Across all our models we have controlled for an array of respondent’s socio-economic and demographic characteristics (Table 1).

**Table 1:** Descriptive statistics of variables used in the analysis

Variables	Total samples (*n* = 259,627)			Sample of women delivered at the public institutions (*n* = 141,028)		
	*n*	Poor (*n* = 130,132)	Non-poor (*n* = 129,495)	*n*	Poor (*n* = 71,446)	Non-poor (*n* = 69,582)
		% (SE)	% (SE)		% (SE)	% (SE)
Dependent variables						
ANC visits						
More than 4	190,898	34.8 (0.17)	65.8 (0.15)	105,615	40.3 (0.23)	64.1 (0.21)
Place of delivery						
Institutional delivery	259,627	66.8 (0.14)	89.8 (0.08)	–	–	–
CFI (12–23 months ago)						
Yes	71,422	56.8 (0.27)	66.2 (0.25)	39,303	62.0 (0.35)	67.7 (0.36)
Infant PNC						
Within 2 days	190,898	23.1 (0.15)	30.3 (0.14)	105,615	26.7 (0.20)	31.3 (0.21)
Child alive						
Yes	259,627	94.3 (0.07)	96.8 (0.05)	141,028	95.1 (0.08)	96.8 (0.07)
Explanatory variables						
Level of FHWE						
Low	86,544	34.2 (0.13)	29.9 (0.12)	36,199	26.7 (0.17)	23.9 (0.17)
Medium	86,607	32.4 (0.14)	34.0 (0.13)	47,620	33.4 (0.18)	32.8 (0.19)
High	86,476	33.3 (0.14)	36.0 (0.13)	57,209	39.9 (0.19)	43.3 (0.20)
Birth order						
1	96,212	31.0 (0.13)	45.4 (0.14)	55,990	35.3 (0.19)	44.6 (0.19)
2	79,670	28.4 (0.13)	35.2 (0.13)	44,600	29.5 (0.18)	35.9 (0.19)
3	41,607	18.3 (0.11)	12.5 (0.09)	21,754	17.4 (0.15)	12.7 (0.13)
3+	42,138	22.3 (0.12)	06.9 (0.07)	18,684	17.8 (0.15)	06.7 (0.10)
Current age of women						
15–19	6699	3.6 (0.05)	02.2 (0.04)	4155	04.1 (0.08)	02.5 (0.06)
20–24	78,177	31.9 (0.14)	32.3 (0.13)	46,061	34.7 (0.19)	35.3 (0.19)
25–29	99,396	36.2 ( 0.14)	41.4 (0.14)	54,319	36.5 (0.19)	41.1 (0.19)
30–34	49,005	17.5 (0.11)	17.6 (0.10)	24,734	16.3 (0.14)	15.8 (0.14)
35–39	19,212	07.6 (0.08)	05.5 (0.06)	8938	6.1 (0.09)	04.5 (0.08)
40–44	5504	2.4 (0.04)	01.0 (0.03)	2242	1.7 (0.05)	00.7 (0.03)
45–49	1634	00.8 (0.03)	00.2 (0.01)	579	00.6 (0.03)	00.1 (0.01)
Educational status of women						
Illiterate	81,087	49.7 (0.15)	12.4 (0.09)	40,278	43.9 (0.19)	12.8 (0.13)
Primary	37,938	18.0 (0.11)	10.5 (0.08)	21,176	18.5 (0.15)	11.7 (0.13)
Secondary	116,646	30.7 (0.13)	58.6 (0.14)	69,756	35.9 (0.19)	63.0 (0.19)
Higher	23,956	1.5 (0.04)	18.5 (0.11)	9818	01.7 (0.05)	12.4 (0.13)
Husband/partner’s educational status						
Illiterate	8181	5.3 (0.06)	01.1 (0.03)	3985	04.6 (0.08)	01.1 (0.04)
Primary	6587	3.4 (0.05)	01.7 (0.04)	3674	03.5 (0.07)	01.9 (0.05)
Secondary	24,511	7.5 (0.08)	11.0 (0.09)	14,253	08.1 (0.11)	11.4 (0.13)
Higher	5783	00.5 (0.02)	04.1 (0.05)	2545	00.6 (0.03)	02.8 (0.06)
Don’t know/missing	214,565	83.4 (0.10)	82.1 (0.10)	116,571	83.2 (0.15)	82.9 (0.15)
Occupation of woman						
Not working	34,426	12.0 (0.09)	14.8 (0.09)	18,750	12.3 (0.13)	14.1 (0.14)
White collar	1382	00.2 (0.01)	00.8 (0.02)	678	00.2 (0.02)	00.6 (0.03)
Agricultural worker	5974	03.1 (0.05)	01.2 (0.03)	3185	02.9 (0.06)	01.2 (0.04)
Service/manual work	3093	01.2 (0.03)	01.1 (0.03)	1742	01.2 (0.04)	01.1 (0.04)
Don’t know/missing	214,752	83.5 (0.11)	82.2 (0.11)	116,673	83.4 (0.15)	82.9 (0.15)
Husband/partner’s occupation						
Not working	1939	00.8 (0.03)	00.7 (0.02)	1109	00.7 (0.03)	00.8 (0.04)
White collar	8945	01.6 (0.04)	05.5 (0.06)	4345	01.7 (0.05)	04.3 (0.08)
Agricultural worker	14,614	06.9 (0.07)	03.6 (0.05)	7992	06.9 (0.09)	03.9 (0.07)
Service/manual work	19,161	07.0 (0.07)	07.9 (0.07)	10,803	07.2 (0.10)	07.9 (0.11)
Don’t know/missing	214,968	83.6 (0.11)	82.2 (0.10)	116,779	83.4 (0.14)	83.0 (0.15)
Caste						
Others	45,019	11.9 (0.09)	26.2 (0.12)	22,160	11.1 (0.12)	22.3 (0.16)
SC	49,051	25.6 (0.13)	17.9 (0.11)	29,879	27.5 (0.17)	22.0 (0.16)
ST	52,199	16.5 (0.11)	5.2 (0.06)	27,546	16.5 (0.14)	05.9 (0.09)
OBC	101,786	41.6 (0.14)	46.5 (0.14)	54,436	40.7 (0.19)	44.8 (0.19)
Don’t know/not reported	11,572	4.3 (0.05)	04.4 (0.06)	7007	04.2 (0.08)	04.9 (0.09)
Religion						
Hindus	187,573	80.6 (0.12)	76.9 (0.12)	106,876	84.6 (0.14)	78.5 (0.16)
Muslims	40,950	16.0 (0.11)	17.0 (0.10)	19,441	12.6 (0.13)	15.3 (0.14)
Christians	20,934	1.6 (0.04)	02.5 (0.03)	9279	01.2 (0.04)	02.2 (0.06)
Others	10,170	1.8 (0.04)	03.6 (0.05)	5432	01.6 (0.05)	03.9 (0.08)
Place of residence						
Rural	198,248	91.9 (0.08)	54.0 (0.14)	110,259	91.4 (0.11)	58.4 (0.19)
Urban	61,379	08.1 (0.08)	45.9 (0.14)	30,769	08.6 (0.11)	41.6 (0.19)
Region						
Northern	48,703	07.6 (0.08)	18.2 (0.11)	29,692	08. (0.11)	21.5 (0.16)
Central	75,645	33.1 (0.14)	21.8 (0.11)	40,356	32.7 (0.18)	21.1 (0.16)
Eastern	54,075	39.0 (0.14)	13.5 (0.09)	29,373	37.4 (0.19)	14.0 (0.14)
North-eastern	37,167	04.8 (0.06)	02.4 (0.04)	18,861	04.7 (0.08)	02.9 (0.07)
Western	18,276	07.8 (0.08)	17.2 (0.10)	8061	07.5 (0.01)	13.9 (0.14)
Southern	25,761	07.6 (0.08)	27.3 (0.12)	14,685	09.3 (0.11)	26.5 (0.17)

*SE* standard error

Below we have explained the BLR model using 4+ ANC visits as an outcome variable. For example, we could define ${4+} \, \text{ANC visits}$ as:

$${y}_{i}\left\{\begin{array}{cc}1& \text{if the }i \text{th woman has made}\, {4+}\, \text{ANC visits} \\ 0& \text{otherwise}\end{array}\right\}.$$

As in [49] Retherford and Choe [50] for the above binary dependent variables (${y}_{i}$), the BLR model takes the following general form:

$$\text{Log} \left(\frac{{P}_{4+\text{ANC visits}}}{{1-{P}}_{4+\text{ANC visits}}}\right)= \text{Logit }\left({{P}}_{4+\text{ANC visits}}\right)={b_0}+{b_1}{x_{1=\text{FHWE}}}+ {b_2}{x_2}+{b_3} {x_3}+\cdots {b_k}{x_k}+{e_k}.$$

$P_{4+\mathrm{ANC visits}}$ is the probability of making 4+ ANC visits,* b*_0_ is the *y* intercept, and *x*_1=FHWE_ is the level of female respondent’s engagement with a FHW, and the term b_1_*x*_1_ is the regression coefficient *x*_1=FHWE._

##### Cox proportion hazard regression model

Next, a Cox proportional hazard model is used to estimate the relationship between FHW’s engagement with mothers and under-five child survival. It is a semi-parametric model which is used in ‘time-to-event’ data with censoring and covariates. The model has used only the rank order of the failure and the censoring times, which is less influenced by the outliers in the failure times [50, 51]. The general form of the model is given as [52]:

$${h_i}(t, X)={h_0}(t)\exp \left\{{\beta^*}X (\text{FHWE})\right\}={h_0}(t)\exp \left\{{\sum_{i=1}^{p}}{\beta_i}{X_{\text{FHWE}}}+{\beta_2}{X_2}+ \cdots+{\beta_k}{X_k})\right\}$$

where* t* represents the survival time, ${h_i}(t, X)$ is the hazard function determined by $X_{1=\mathrm{FHWE}}$ and controlled for covariates (*x*_2_,…,*x*_*k*_), and the coefficients ($\beta$_1_, $\beta$_2_,…,$\beta$_*k*_) measure the impact (i.e. the effect size) of covariates.

The term ${h_0}(t)$ is called the baseline hazard. It corresponds to the value of the hazard if all the $X_i$ are equal to zero (the quantity exp (0) equals 1). The ‘*t*’ in* h*(*t*) reminds us that the hazard may vary over time [32].

## Results

### Descriptive statistics

Table 1 presents the descriptive statistics for all the variables used in this study, disaggregated by the household’s economic status (poor and non-poor). The household’s economic status is based on the wealth index that is available in the NFHS dataset, where households are classified into five wealth quintiles, constructed using assets owned by households and applying principal components analysis [3]. We classify the first two categories (poorest and poor households) as being in the category ‘poor’, and classify households in the middle, rich and richest as being ‘non-poor’ [11, 53].

From the descriptive statistics for the full sample presented in Table 1, we observe that across all our five outcome variables, women from non-poor households have better MCH care and child health outcomes. Women from non-poor households received relatively better ANC care. In particular, 65.8% had 4+ antenatal visits, in comparison to 34.8% for women from poor households. Approximately, 89.8% of women from non-poor households gave birth at health facilities relative to 66.8% among women from poor households, and their children also received more full immunisation (66.2% compared to 56.6% for poor women). Furthermore, although postnatal care of children was generally low in the sample, a higher proportion of children from non-poor households were PNC (30.3%) relative to children from poor households (23.1%). We also observe that there were fewer under-five child deaths (3.2%) in the non-poor sample relative to 5.7% among poor women. Further, engagement with FHWs was also greater among married women from non-poor households (36%) relative to women from poor households (33.3%). Women with higher-order births (3 or more children) are more likely to be present in poor households (22.3%) compared to 6.9% among non-poor households. However, in the sample of women who gave birth in public institutions, the economic differences in MCH care and outcome indicators and engagement with FHWs is slightly lower.

The sample distribution for other background characteristics by the economic status of women who delivered in all facilities and public institutions are as expected. In India, caste is considered to be an important marker of social disadvantage, and the Indian government has introduced a policy of affirmative action, for individuals from social and economic backward castes and tribes [called Scheduled Castes (SCs), STs, and Other Backward Castes (OBCs)]. Among the poor, 41.6% of the sample are from OBCs, 25.6% are from SCs, and 16.5% are from STs and 16.3% are from others. This distribution is 46.5%, 17.9%, 9.4% and 26.2%, respectively, among the non-poor. In terms of religion, majority of to the sample are Hindus (poor: 80.6%, non-poor: 76.9%) followed by Muslims (poor: 16.0%, non-poor: 17.05%). About 91.9% of poor and 54% of non-poor women live in rural areas.

### Association between FHWE and MCH services

Table 2 shows unadjusted and adjusted estimates of odds ratios from the BLR model. The adjusted estimates show the association between the MCH services and the level of FHWE for the disaggregate samples of poor and non-poor women controlling for an array of socio-demographic characteristics. The unadjusted results show that among economically poor women having higher engagement with FHWs are 8.02 times (*p* < 0.05) more likely to access 4 and more ANC visits, relative to those with low FHW engagement. Similarly, among females from poor households, the likelihood of institutional delivery is 2.80 times (*p* < 0.05) greater for women with higher FHW engagement than for those with low FHW engagement. Furthermore, among poor households, having a high level of engagement with maternal engagement with FHWs is associated with 2.88 times (*p* < 0.01) higher likelihood of CFI and 4.65 times (*p* < 0.05) higher likelihood of PNC (within 2 days of delivery), compared to women with those who have low-level of FHWE. Although the influence of FHW engagement on MCH services among non-poor women is slightly low compared to poor women, within the sample of non-poor women, the utilisation of MCH services is almost two times higher among those with higher FHW engagement compared to their counterparts. Differences in MCH services by the level of FHW engagement do not change in adjusted estimates in both the poor and non-poor samples.

**Table 2:** Odds ratio estimates from binary logistic regression model showing mother and child (0–5 years) health care by levels of women’s FHWE among the poor and non-poor households in India, 2015–2016

Predictor variables	Antenatal care (4 or > 4 times)			Place of delivery (all institutions)			Child full immunisation (12–23 months)			Infant postnatal care (within 2 days of delivery)		
	Odds ratio (SE^a^)			Odds ratio (SE)			Odds ratio (SE)			Odds ratio (SE)		
	Poor (*n* = 90,521)	Non-poor (*n* = 100,377)	Total (n = 190,898)	Poor (*n* = 130,132)	Non-poor (*n* = 129,495)	Total (*n* = 259,627)	Poor (*n* = 36,995)	Non-poor (*n* = 34,427)	Total (*n* = 71,422)	Poor (*n* = 90,521)	Non-poor (*n* = 100,377)	Total (*n* = 190,898)
Panel A (unadjusted)												
Level of FHWE												
Low												
Medium	2.448* (0.06)	1.704* (0.03)	1.855* (0.02)	1.805* (0.03)	1.729* (0.04)	1.801* (0.02)	1.784* (0.05)	1.444* (0.04)	1.627* (0.03)	1.971* (0.06)	1.554* (0.03)	1.723* (0.03)
High	8.023* (0.18)	2.297* (0.04)	3.375* (0.04)	2.803* (0.04)	2.684* (0.06)	2.746* (0.03)	2.882* (0.08)	2.109* (0.06)	2.505* (0.05)	4.647* (0.11)	2.713* (0.05)	3.334* (0.05)
Panel B (adjusted)												
Level of FHWE												
Low												
Medium	2.174* (0.05)	1.766* (0.03)	1.771* (0.03)	1.678* (0.03)	1.616* (0.04)	1.659* (0.02)	1.717* (0.05)	1.429* (0.04)	1.586* (0.03)	1.95*** (0.06)	1.567* (0.03)	1.706* (0.03)
High	6.367* (0.16)	2.489* (0.04)	3.305* (0.05)	2.538* (0.04)	2.543* (0.07)	2.531* (0.03)	2.692* (0.08)	2.067* (0.06)	2.406* (0.05)	4.63*** (0.12)	2.876* (0.05)	3.480* (0.05)
Birth order												
1												
2	0.747* (0.02)	0.777* (0.01)	− 0.277* (0.01)	0.582* (0.01)	0.519* (0.01)	0.554* (0.01)	0.827* (0.03)	0.871* (0.02)	0.846* (0.02)	0.965 (0.02)	0.958* (0.02)	0.957* (0.01)
3	0.588* (0.02)	0.597* (0.01)	− 0.557* (0.02)	0.464* (0.01)	0.356* (0.01)	0.411* (0.01)	0.769* (0.03)	0.696* (0.03)	0.729* (0.02)	0.911* (0.02)	0.949* (0.02)	0.918* (0.02)
3+	0.426* (0.01)	0.421* (0.01)	− 0.962* (0.02)	0.389* (0.01)	0.273* (0.01)	0.328* (0.01)	0.718* (0.03)	0.622* (0.03)	0.653* (0.02)	0.952 (0.03)	0.881* (0.03)	0.874* (0.02)
Current age of woman												
15–19												
20–24	0.955 (0.04)	1.054 (0.05)	0.026 (0.03)	0.969 (0.04)	0.909 (0.06)	0.999 (0.04)	1.088 (0.07)	1.009 (0.08)	1.066 (0.05)	1.074 (0.05)	0.959 (0.02)	1.039 (0.03)
25–29	1.043 (0.04)	1.302* (0.06)	0.221* (0.03)	1.042 (0.04)	1.113 (0.09)	1.164* (0.04)	1.159* (0.07)	1.152* (0.09)	1.186* (0.06)	1.119* (0.05)	0.999 (0.02)	1.098* (0.04)
30–34	1.143* (0.05)	1.606* (0.08)	0.409* (0.03)	1.135* (0.05)	1.577* (0.13)	1.416* (0.06)	1.159* (0.08)	1.249* (0.11)	1.246* (0.07)	1.119* (0.06)	1.008 (0.05)	1.117* (0.04)
35–39	1.195* (0.06)	1.720* (0.09)	0.454* (0.04)	1.004 (0.05)	1.764* (0.16)	1.326* (0.06)	1.090 (0.09)	1.577* (0.16)	1.314* (0.08)	1.144* (0.06)	1.198* (0.07)	1.239* (0.05)
40–44	1.097 (0.08)	1.641* (0.13)	0.355* (0.05)	0.898* (0.05)	1.346* (0.15)	1.130* (0.06)	1.109 (0.13)	1.385* (0.25)	1.264* (0.12)	1.141* (0.08)	1.189* (0.09)	1.198* (0.06)
45–49	0.931 (0.11)	1.369* (0.22)	0.159* (0.09)	0.758* (0.06)	0.929 (0.16)	0.918 (0.07)	1.380* (0.23)	1.156 (0.50)	1.403* (0.22)	0.911 (0.10)	1.218 (0.19)	0.999 (0.09)
Education status of women												
Illiterate												
Primary	1.556* (0.04)	1.290* (0.04)	0.453* (0.02)	1.252* (0.02)	1.194* (0.04)	1.272* (0.02)	1.269* (0.04)	1.317* (0.06)	1.336* (0.03)	1.082* (0.03)	1.069* (0.04)	1.123* (0.02)
Secondary	1.724* (0.04)	1.713* (0.04)	0.741* (0.01)	1.837* (0.03)	2.133* (0.05)	2.263* (0.03)	1.443* (0.04)	1.513* (0.06)	1.604* (0.03)	1.061* (0.02)	1.171* (0.03)	1.221* (0.02)
Higher	2.146* (0.14)	2.357* (0.07)	1.145* (0.02)	3.203* (0.24)	4.685* (0.21)	5.587* (0.20)	1.557* (0.15)	1.736* (0.08)	1.908* (0.07)	1.152* (0.08)	1.333* (0.04)	1.447* (0.03)
Education status of husband/partner												
Illiterate												
Primary	1.044 (0.06)	1.135 (0.09)	0.106* (0.05)	1.132* (0.05)	0.939 (0.09)	1.077* (0.04)	1.121 (0.09)	0.983 (0.13)	1.056 (0.07)	1.067 (0.06)	1.001 (0.09)	1.052 (0.05)
Secondary	1.077 (0.05)	1.186* (0.09)	0.192* (0.04)	1.272* (0.05)	1.324* (0.10)	1.331* (0.04)	1.206* (0.08)	1.372* (0.15)	1.288* (0.07)	1.019 (0.05)	1.057 (0.08)	1.076* (0.04)
Higher	1.038 (0.12)	1.314* (0.11)	0.343* (0.05)	1.593* (0.19)	1.689* (0.17)	1.803* (0.12)	1.173 (0.21)	1.166 (01.4)	1.168* (0.09)	1.030 (0.13)	0.975 (0.08)	1.017 (0.05)
Don't know/missing	1.061 (0.23)	1.077 (0.23)	0.129 (0.15)	1.230 (0.21)	2.185* (0.58)	1.565* (0.22)	1.209 ( 0.39)	1.289 (0.45)	1.239 (0.29)	1.073 (0.24)	0.922 (0.19)	1.013 (0.16)
Occupational status of woman												
Not working												
White collar	0.686* (0.14)	0.771* (0.07)	− 0.281* (0.08)	1.089 (0.19)	1.013 (0.17)	1.076 (0.13)	3.159* (1.09)	1.085 (0.17)	1.323* (0.19)	1.258 (0.24)	1.111* (0.09)	1.119* (0.08)
Agricultural worker	0.919 (0.05)	1.107 (0.08)	− 0.031 (0.04)	0.857* (0.04)	0.712* (0.06)	0.816* (0.03)	0.946 (0.07)	1.116 (0.13)	0.970 (0.06)	1.113* (0.06)	1.226* (0.08)	1.147* (0.05)
Service/manual work	1.130 (0.09)	1.020 (0.07)	− 0.066 (0.05)	0.810* (0.05)	0.691* (0.06)	0.771* (0.04)	1.322* (0.15)	0.845 (0.10)	1.047 (0.09)	1.205* (0.09)	1.124* (0.07)	1.156* (0.06)
Don’t know/missing	0.996 (0.17)	0.752 (0.13)	− 0.159 (0.12)	0.801* (0.11)	0.581* (0.13)	0.695* (0.08)	1.045 (0.28)	1.052 (0.03)	1.051 (0.19)	1.245 (0.22)	1.311 (0.22)	1.277* (0.15)
Occupational status of husband/ partner												
Not working												
White collar	0.980 (0.12)	1.137 (0.09)	0.129* (0.07)	1.052 (0.09)	0.956 (0.13)	1.091 (0.08)	0.959 (0.16)	1.447* (0.20)	1.255* (0.13)	1.289* (0.16)	1.068 (0.09)	1.149* (0.08)
Agricultural worker	1.003 (0.11)	0.933 (0.08)	− 0.017 (0.07)	0.956 (0.08)	0.859 (0.11)	0.910 (0.06)	1.042 (0.15)	1.099 (0.16)	1.091 (0.12)	1.027 (0.11)	0.923 (0.08)	0.961 (0.07)
Service/manual work	1.092 (0.11)	1.227* (0.11)	0.163* (0.07)	1.128 (0.09)	0.891 (0.11)	1.053 (0.07)	1.232 (0.18)	1.614* (0.22)	1.406* (0.14)	1.320* (0.15)	1.166* (0.09)	1.221* (0.08)
Don’t know/missing	0.984 (0.20)	1.427* (0.25)	0.188 (0.13)	1.143 (0.17)	0.825 (0.19)	1.042 (0.13)	0.947 (0.25)	1.129 (0.32)	1.029 (0.20)	0.937 (0.22)	0.919 (0.16)	0.923 (0.13)
Caste												
Others												
SC	0.723* (0.02)	0.839* (0.02)	− 0.306* (0.02)	0.973 (0.02)	0.737* (0.02)	0.816* (0.02)	0.958 (0.04)	1.043 (0.04)	0.982 (0.03)	1.069* (0.03)	0.998 (0.02)	0.969* (0.02)
ST	0.807* (0.03)	0.781* (0.03)	− 0.259* (0.02)	0.629* (0.02)	0.488* (0.02)	0.517* (0.01)	0.754* (0.04)	0.985 (0.06)	0.793* (0.03)	1.096* (0.04)	0.906* (0.03)	0.954* (0.02)
OBC	0.626* (0.02)	0.769* (0.01)	− 0.377* (0.02)	1.075* (0.02)	0.884* (0.02)	0.948* (0.02)	0.939 (0.04)	1.058* (0.03)	0.992 (0.02)	1.077* (0.03)	0.966* (0.02)	0.972* (0.01)
Don’t know/missing	1.101* (0.05)	1.029 (0.04)	0.029 (0.03)	0.874* (0.03)	1.171* (0.06)	0.899* (0.03)	1.013 (0.07)	1.130* (0.07)	1.052 (0.04)	0.867* (0.04)	0.800* (0.03)	0.805* (0.02)
Religion												
Hindus												
Muslims	1.048* (0.03)	1.140* (0.02)	0.118* (0.02)	0.556* (0.01)	0.660* (0.02)	0.591* (0.01)	0.804* (0.03)	0.839* (0.03)	0.829* (0.02)	1.016 (0.03)	1.148* (0.02)	1.105* (0.02)
Christians	1.057 (0.07)	0.885* (0.04)	− 0.013 (0.04)	0.589* (0.03)	0.919 (0.08)	0.770* (0.03)	1.198* (0.12)	1.099 (0.09)	1.169* (0.07)	0.908 (0.06)	1.039 (0.05)	1.007 (0.04)
Others	1.429* (0.08)	1.391* (0.06)	0.347* (0.03)	0.574* (0.03)	1.287* (0.08)	0.814* (0.03)	1.358* (0.12)	1.741* (0.13)	1.620* (0.09)	1.125* (0.07)	1.490* (0.05)	1.404* (0.04)
Place of residence												
Rural												
Urban	1.576* (0.05)	1.451* (0.02)	0.607* (0.01)	1.243* (0.03)	1.375* (0.02)	1.611* (0.02)	1.064 (0.05)	0.957* (0.02)	1.085* (0.02)	1.096* (0.03)	1.057* (0.02)	1.164* (0.01)
Region												
Northern												
Central	0.551* (0.02)	0.694* (0.02)	− 0.544* (0.02)	0.553* (0.02)	0.663* (0.02)	0.574* (0.01)	0.937 (0.04)	0.789* (0.03)	0.776* (0.02)	1.064* (0.04)	1.221* (0.03)	1.073* (0.02)
Eastern	0.829* (0.03)	1.157* (0.03)	− 0.186* (0.02)	0.484* (0.01)	0.823* (0.03)	0.512* (0.01)	1.734* (0.08)	1.356* (0.06)	1.361* (0.04)	0.870* (0.03)	0.885* (0.02)	0.794* (0.02)
North-eastern	0.946* (0.04)	1.313* (0.06)	− 0.056* (0.03)	0.471* (0.02)	0.796* (0.05)	0.483* (0.02)	0.740* (0.05)	0.634* (0.05)	0.611* (0.03)	0.976 (0.05)	0.712* (0.04)	0.762* (0.03)
Western	2.299* (0.09)	2.265* (0.06)	0.765* (0.02)	0.952* (0.03)	2.083* (0.08)	1.339* (0.03)	0.731* (0.04)	0.544* (0.02)	0.579* (0.02)	1.157* (0.05)	0.841* (0.20)	0.881* (0.02)
Southern	3.451* (0.15)	2.780* (0.06)	1.041* (0.02)	1.448* (0.06)	3.395* (0.13)	2.319* (0.07)	1.118* (0.07)	0.807* (0.03)	0.869* (0.03)	1.003 (0.04)	0.982 (0.02)	0.959* (0.02)

*FHWE* Frontline Health Worker Interaction, *CFI* Child Full Immunisation

*p < 0.05

^a^SE represents standard error in the parentheses

**Fig. 1:**
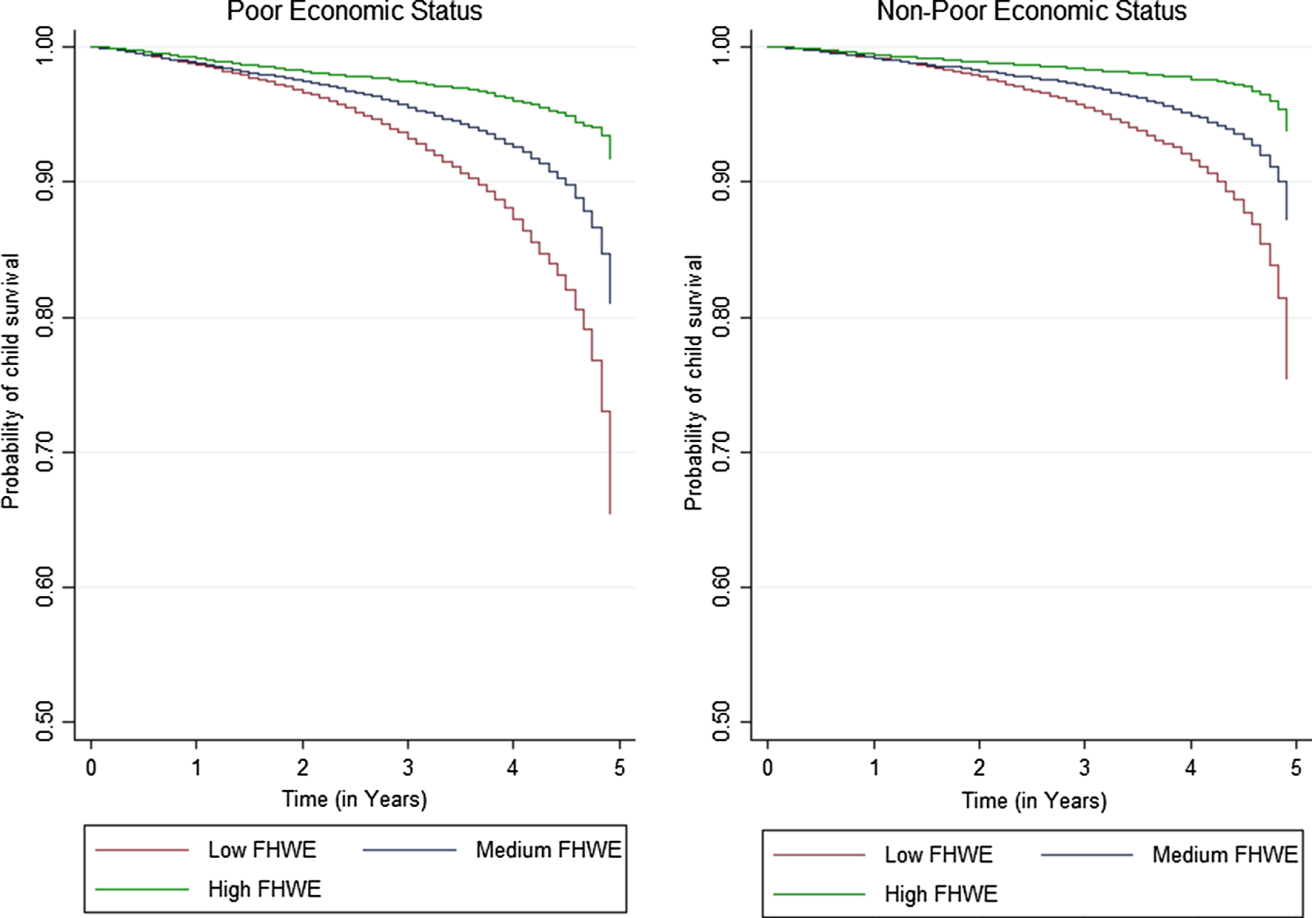
Kaplan–Meier child survival estimates by level of mother’s FHWE among the poor and non-poor households in India, 2015–2016

Table 3 presents the unadjusted and adjusted hazard ratio (HR) from the Cox proportional hazards regression model. The results show that relative to those with a low level of engagement with FHWs, a high level of engagement with FHWs reduces the relative risk of under-five child deaths by about five times (HR = 0.220, *p* < 0.01) for poor women and by four times (*p* < 0.01) for non-poor women. These results are in line with the Kaplan–Meier's survival estimates. The Kaplan–Meier’s probability of survival plots suggests significant survival differences by the level of FHW engagement among the samples of both poor and non-poor women. However, it is notable that engagement with FHWs is making a slightly greater difference in child survival times for poor women relative to non-poor women. In other words, poor women benefit relatively more from engagement with FHW than non-poor women (Fig. 1).

**Table 3:** Hazard ratio estimates from the Cox proportional hazard regression model: child survival outcome by mother’s level of FHWE among the poor and non-poor households in India, 2015–2016

Predictor variables	Hazard ratio (SE)		
	Poor (*n* = 130,132)	Non-poor (*n* = 129,495)	Total (*n* = 259,627)
Panel A (unadjusted)			
Level of FHWE			
Low			
Medium	0.569* (0.02)	0.589* (0.03)	0.561* (0.02)
High	0.220* (0.01)	0.254* (0.02)	0.227* (0.01)
Panel B (adjusted)			
Level of FHWE			
Low			
Medium	0.572* (0.03)	0.599* (0.03)	0.585* (0.02)
High	0.223* (0.01)	0.251* (0.02)	0.236* (0.01)
Birth order			
1			
2	0.937 (0.06)	1.187* (0.08)	1.045 (0.05)
3	1.091 (0.07)	1.389* (0.12)	1.233* (0.06)
3+	1.318* (0.09)	2.433* (0.23)	1.657* (0.09)
Current age of woman			
15–19			
20–24	0.535* (0.06)	0.657* (0.13)	0.548* (0.05)
25–29	0.463* (0.05)	0.525* (0.10)	0.451* (0.04)
30–34	0.438* (0.05)	0.430* (0.09)	0.401* (0.04)
35–39	0.448* (0.06)	0.487* (0.10)	0.425* (0.05)
40–44	0.465* (0.07)	0.553* (0.14)	0.451* (0.06)
45–49	0.539* (0.09)	0.995 (0.29)	0.577* (0.09)
Education status of women			
Illiterate			
Primary	0.998 (0.05)	0.912 (0.08)	0.959 (0.11)
Secondary	0.798* (0.04)	0.770* (0.06)	0.748* (0.03)
Higher	0.506* (0.12)	0.459* (0.05)	0.413* (0.04)
Education status of husband/partner			
Illiterate			
Primary	0.851 (0.11)	1.091 (0.27)	0.911 (0.11)
Secondary	0.783* (0.09)	0.922 (0.19)	0.834* (0.08)
Higher	0.651 (0.24)	0.546* (0.15)	0.534* (0.10)
Don't know/missing	0.346* (0.16)	0.480 (0.32)	0.369* (0.14)
Occupational status of woman			
Not working			
White collar	0.719 (0.36)	1.104 (0.36)	0.984 (0.27)
Agricultural worker	0.928 (0.12)	0.875 (0.21)	0.906 (0.10)
Service/manual work	0.927 (0.18)	1.487* (0.31)	1.128 (0.16)
Don’t know/missing	1.493 (0.57)	1.184 (0.67)	1.442 (0.46)
Occupational status of husband/partner			
Not working			
White collar	0.764 (0.21)	1.267 (0.46)	0.880 (0.18)
Agricultural worker	0.760 (0.17)	1.482 (0.53)	0.917 (0.10)
Service/manual work	0.810 (0.18)	1.301 (0.45)	0.939 (0.18)
Don’t know/missing	1.340 (0.49)	2.107 (1.13)	1.528 (0.46)
Caste			
Others			
SC	1.138 (0.09)	1.243* (0.11)	1.228* (0.07)
ST	1.349* (0.01)	1.435* (0.17)	1.445* (0.09)
OBC	1.023 (0.08)	1.119 (0.08)	1.090* (0.06)
Don’t know/missing	1.110 (0.13)	1.172 (0.17)	1.169* (0.11)
Religion			
Hindus			
Muslims	0.992 (0.06)	0.791* (0.06)	0.919* (0.04)
Christians	0.855 (0.09)	1.403* (0.19)	1.024 (0.08)
Others	0.866 (0.12)	0.917 (0.13)	0.867 (0.08)
Place of residence			
Rural			
Urban	0.882 (0.07)	0.824* (0.05)	0.786* (0.03)
Region			
Northern			
Central	1.414* (0.09)	1.457* (0.10)	1.462* (0.07)
Eastern	0.941 (0.07)	0.784* (0.08)	0.948 (0.05)
North-eastern	0.936 (0.09)	0.831 (0.10)	0.952 (0.07)
Western	0.897 (0.11)	0.755* (0.09)	0.830* (0.07)
Southern	1.186 (0.15)	0.908 (0.90)	0.987 (0.08)

*SE* standard error, *FHWE* Frontline Health Worker Interaction

*p < 0.05

### Association between socio-demographic factors and MCH care and outcomes

In addition to maternal engagement with FHWs, MCH care service utilisation are also influenced by an array of socio-economic and demographic characteristics. For instance, mothers with less than two children were more likely to have better health care utilisation (such as giving birth at health facilities, ANC visits, higher levels of postnatal care, prescribed set of child immunisation) and child survival rates, relative to mothers with more than two children. The utilisation of MCH care and child survival varies by the social and religious affiliation, and is also higher among urban residents. In terms of regional disparities, women from Southern India have a higher probability of utilisation of MCH services relative to women from other regions (Tables 2, 3). These findings are in line with previous studies that documented determinants of maternal and child health care [7–9, 54, 55].

### Robustness checks

Following [46], we have used sub-sample analyses to check the robustness of the relationship between the level of FHW engagement and utilisation of MCH care and child survival in India. We have categorised women who delivered in public health hospitals and those from rural areas as two separate sets of sub-samples (Additional file 1: Fig S1; Additional file 2: Fig S2; Additional file 3: Fig S3; Additional file 4: Fig S4; Additional file 5: Fig S5).

### Results based on the sub-sample of women who delivered in public institutions

Table 5 demonstrates the unadjusted and adjusted effects of engagement with FHWs on the utilisation of MCH services for the sub-sample of women who delivered only in public health care institutions. After controlling for all other potential socio-economic and demographic confounders, the adjusted probabilities suggest that differences in the likelihood of MCH care utilisation by the level of FHW engagement is not statistically different in the sample of women who delivered in public institutions relative to the overall sample. Specifically, we observe that poor women with high levels of FHW engagement have a 6.57 times (*p* < 0.01) higher likelihood of having four or more ANC visits and they are 2.59 times (*p* < 0.01) more likely to have had an institutional delivery in public health facilities. Moreover, their children have a 2.38 times (*p* < 0.01) higher likelihood of CFI and they are 4.34 times (*p* < 0.01) more likely to receive PNC relative to those with low levels of FHW engagement. Note that high levels of FHW engagement is also beneficial for non-poor women, who are also nearly two times more likely to access MCH services if they have a high level of FHW engagement. In keeping with the analyses based on the full sample, analyses of the sub-sample of women who delivered in public health institutions also show that after controlling for all socio-economic and demographic characteristics, the differences in the odds of obtaining MCH care by the level of engagement with FHWs is higher among poor compared to the non-poor women.

### Results based on women from rural areas

The analyses of the sub-sample of women from rural areas further strengthen our argument that engagements with FHWs help women in accessing MCH care and child survival outcomes. We observe that compared to poor women with low FHWI, those with high FHWI in rural areas have 8.73 times (*p* < 0.01) higher likelihood of having more than four ANC visits and they are 2.58 times (*p* < 0.01) more likely to have institutional delivery. Further, their children have 2.66 times (*p* < 0.01) higher probability of CFI and they are 4.81 times (*p* < 0.01) more likely to seek PNC relative to those with low FHW engagement. Non-poor women also have about two to three times higher probability of accessing MCH services if they have a high level of FHWE. Thus, once again the results suggest that after controlling for all the socio-economic and demographic characteristics, differences in the odds of obtaining MCH care by the level of engagement with FHWs are higher among poor compared to the non-poor (Table 6).

Similarly, the analyses of child survival outcomes from the sub-sample of women from rural areas show that the differences in the hazard of death by the level of mother’s engagement with FHWs for an under-five child are significantly low in women with a higher FHWE compared to their counterparts. Such patterns also not vary by their economic status net of other socio-economic and demographic characteristics (Table 5).

## Discussion

The focus of previous studies on the issue of ‘influence of FHWs on MCH care and outcomes’ has been on the relationship between women’s engagement with CHWs in general and ASHAs in particular. Furthermore, these studies have typically focused on case studies, observational or experimental studies conducted in a specific geographical area mostly using small samples [13, 14, 18, 30, 31, 41, 45]. In this study, we have addressed a research gap by using a nationally representative sample and adopting a comprehensive definition of FHWs to investigate the influence of the level of women’s engagement with FHWs on various MCH care and outcome indicators. Our analyses find that the level of women’s engagement with FHWs is positively associated with MCH care and outcomes, irrespective of the socio-economic and demographic characteristics of the women. The findings also suggest that poor women have a greater chance of benefiting from the interaction with FHWs compared to non-poor. The analysis of sub-samples of women who delivered only in public health facilities and rural women is consistent with previous evidence and supports the robustness of our study. Thus, our study supports the argument of *Close To Community (CTC)* potential of FHWs for expanding the utilisation of MCH services especially among the poor women, which often stand at the fringes of the health system [7, 53, 55]. Considering that except MPHWs, ANMs, ASHAs and AWW are female health workers, thus created a great comfort zone for women to reaching out to FHWs and discuss their MCH care issues, especially in patriarchal societies like South Asia [56]. Our findings are in keeping with previous studies that examined the role of FHWs in improving MCH care globally [21, 22, 24, 27, 41], and in India [13, 14, 18, 30, 31, 41, 45].

Intervention through demand-side determinants and demand-side financing are well discussed and debated strategies in policy and programmes to ensure equity in MCH care and outcomes [5, 8, 10, 11, 34]. However, enhancing engagement with FHWs can be just as cost-effective, results-driven and are shown to be a proven intervention for bringing equity in MCH care and outcomes [28, 56, 57]. However, previous studies have reported that factors such as a lack of skills, low qualifications, the multitasking nature of their job, low incentives, and low recognition is discouraging many FHWs from remaining in this profession [40, 42, 44, 45]. Addressing the shortage of FHWs, enhancing skills and the use of innovative tools to track pregnancies and MCH care can be a more cost-effective strategy to bring equity in MCH services utilisation and outcomes in India and other developing countries [19, 26, 32]. Findings and recommendations from previous studies in other geographical contexts also support our recommendations [23, 56].

### Limitations of the study and scope of future research

Our study is unable to document three aspects of the role of FHWs in improving MCH care and outcomes in the Indian context. First, we are unable to comment on the professional skills and expertise of the FHWs as the NFHS dataset does not provide this information. Second, we are unable to identify reasons for the non-interaction of women with FHWs. Third, although information in the NFHS data on the index of level of engagement with FHWs covers the quantity and quality of interaction, it is not a sufficiently comprehensive measure to capture quality in a true sense. Fourth, considering NFHS do not ask questions on interaction of women with each of FHWs (i.e. ANMs, ASHAs, AWW and MPHWs) separately. Thus, it is not possible to document the effect of women’s interaction with FHWs on MCH care and outcomes, separately for ANMs, ASHAs, AWW and MPHWs and also by the gender of health worker. Thus, future rounds of NFHS must enhance the module on the interaction of FHWs with respondents, and collect comprehensive information on the quantity and quality of their engagement with FHWs to identify the constraints facing those who do not engage with FHWs.

## Conclusion

Our findings have identified FHWs as an integral part of the health system with the potential to make a significant difference to the health of women and children from poor households. FHWs alongside a high-quality public healthcare infrastructure can be efficient forms of supply-side pathways to address under-utilisation of MCH services and improve maternal and child health outcomes, especially among economically disadvantaged people, particularly those living in areas with poor infrastructure [58]. FHWs can work as an interface between people and programmes, and can play a crucial role in motivating people to access health services. FHWs also help in the identification and tracking of potentially vulnerable groups. There is a need to understand barriers in the lower engagement of pregnant women with FHWs. The role of FHWs in the government health system needs to be enhanced by improving skills, working environment, and greater financial incentives.

### Supplementary Information

**Additional file 1: Fig S1.** Maternal health care by FHWE for Poor and Non-Poor women in India, 2015–2016.

**Additional file 2: Fig S2.** Child health care and outcomes by FHWE for Poor and Non-Poor women in India, 2015–2016.

**Additional file 3: Fig S3.** ANCs utilisation by FHWE for Poor and Non-poor women delivered in public health institutions in India, 2015–2016.

**Additional file 4: Fig S4.** Child health care and outcomes by FHWE for Poor and Non-poor women delivered in public health institutions in India, 2015–2016.

**Additional file 5: Fig S5.** Kaplan–Meier survival estimates by level of FHWE for the Poor and Non-Poor women delivered in public health institutions in India, 2015–2016.

**Additional file 6: Table S1.** Descriptive statistics of background characteristics by maternal and child health outcomes in India, 2015–2016. **Table S2.** Descriptive statistics of background characteristics by maternal and child health outcomes among women delivered in public health institutions in India, 2015–2016. **Table S3.** Descriptive statistics of background characteristics by maternal and child health outcomes in India, 2015–2016. **Table S4.** Odds ratio by using Binary Logistic Regression (BLR) model of mother and child (0–5 years) health outcomes by levels of FHWE among the poor and non-poor women delivered in public health institutions in India, 2015–2016. **Table S5.** Hazard ratio by using Cox Proportional Hazard regression model of mother and child (0–5 years) health outcomes by FHWE Level, among the poor and non-poor women delivered in public health institutions in India, 2015–2016. **Table S6.** Odds ratio by using Binary Logistic Regression (BLR) model of mother and child (0–5 years) health outcomes by levels of FHWE among the poor and non-poor of rural women in India, 2015–2016. **Table S7.** Hazard ratio by using Cox Proportional Hazard regression model of child survival outcome by FHWE Level, among the poor and non-poor of rural women in India, 2015–2016.

## Data Availability

The datasets used in this study are publically available at https://dhsprogram.com/what-we-do/survey/survey-display-355.cfm.

## Appendix

See Tables 4, 5 and 6.

**Table 4:** List of indicators considered for the Frontline Health Worker Engagement Index, 2015–2016, [AIC: 0.0389, Coefficient: 0.9409]

S. no	Indicators		1^a^	0^b^
1.	Pregnancy registered		Yes = 1	No = 0
2.	Receives mother and child protection card after registration		Yes = 1	No = 0
3.	Prenatal care	ANM/nurse/mid-wife/LHV	Yes = 1	No = 0
4.		Community village health worker (CHW)	Yes = 1	No = 0
5.		Anganwadi (AWW)/ICDS worker	Yes = 1	No = 0
6.		ASHA	Yes = 1	No = 0
7.	Assistance provided by	ANM/nurse/mid-wife/LHV	Yes = 1	No = 0
8.		Other health personnel	Yes = 1	No = 0
9.	Antenatal care (ANC)	Public Anganwadi/ICDS Centre	Yes = 1	No = 0
10.		Public village clinic by ANM	Yes = 1	No = 0
11.		Other public village health facility	Yes = 1	No = 0
12.	Pregnant woman met with	ANM or LHV in last 3 months	Yes = 1	No = 0
13.		AWW/ASHA/other CHW in last 3 months	Yes = 1	No = 0
14.	Person met with the pregnant woman	AWW	Yes = 1	No = 0
15.		ASHA	Yes = 1	No = 0
16.		MPW	Yes = 1	No = 0
17.		Other health worker	Yes = 1	No = 0
18.	During the last 3 months of pregnancy	Visited health facility for self or child	Yes = 1	No = 0
19.		Person met during most recent contact	$$\left[\begin{array}{c}\text{ANM}\\ \text{LHV}\\ \text{AWW}\\ \text{ASHA}\\ \text{MPW}\end{array}\right]$$ = 1	Other = 0
20.		Met with ANM/LHV/ASHA, Anganwadi worker	Yes = 1	No = 0
21.	Person who arranged transport	ANM	Yes = 1	No = 0
22.		Health worker	Yes = 1	No = 0
23.		AWW	Yes = 1	No = 0
24.		ASHA	Yes = 1	No = 0
25.	During pregnancy women received benefits from Anganwadi/ICDS Centre		Yes = 1	No = 0
26.	Benefits received during pregnancy from Anganwadi/ICDS Centre	Supplementary food/nutrition	Yes = 1	No = 0
27.		Health check-ups	Yes = 1	No = 0
28.		Health and nutrition education	Yes = 1	No = 0
29.	Child received benefits from Anganwadi/ICDS Centre in last 12 months after birth		Yes = 1	No = 0
30.	Child received immunisation through Anganwadi/ICDS Centre in last 12 months after birth		Yes = 1	No = 0
31.	Frequency of services received from Anganwadi/ICDS Centre, in last 12 months after birth	Food	$$\left[\begin{array}{c} \text{almost daily}/\\ \text{at least once a week}\end{array}\right]$$= 1	$$\left[\begin{array}{c}\text{Not at all}/\\ \text{less often/don't know}\end{array}\right]$$= 0
32.		Health check-up	[at least once a month] = 1	$$\left[\begin{array}{c}\text{Not at all}/\\ \text{less often/don't know}\end{array}\right]$$= 0
33.		Childhood care or pre-school	[regularly/occasionally] = 1	$$\left[\begin{array}{c}\text{Not at all}/\\ \text{don't know}\end{array}\right]$$=0
34.		Weight measured	$$\left[\begin{array}{c} \text{atleast once a months}/\\ \text{at least once in 3 months}\end{array}\right]$$=1	$$\left[\begin{array}{c}\text{Not at all}/\\ \text{less often/don't know}\end{array}\right]$$=0
35.	After child weighted, mother received counselling from Anganwadi/ICDS Centre		Yes = 1	No/Don’t know = 0
36.	While breastfeeding, mother received benefits from Anganwadi/ICDS Centre		Yes = 1	No/Didn’t breastfeed = 0
37.	Benefits received while breastfeeding from Anganwadi/ICDS Centre	Supplementary food	Yes = 1	No = 0
38.		Health check-ups	Yes = 1	No = 0
39.		Health and nutrition education	Yes = 1	No = 0
40.	Place met ANM/LHV/ASHA/AWW/CHW with pregnant woman		Home/Both = 1	Elsewhere = 0
41.	Told about	Pregnancy complications to the pregnant woman or family members	Yes = 1	No = 0
42.		Where to go for the pregnancy complications	Yes = 1	No = 0
43.		Vaginal bleeding	Yes = 1	No = 0
44.		Convulsions	Yes = 1	No = 0
45.		Prolonged labour	Yes = 1	No = 0
46.		Severe abdominal pain	Yes = 1	No = 0
47.		High blood pressure	Yes = 1	No = 0
48.	Receive advice	Institutional delivery	Yes = 1	No = 0
49.		Cord care	Yes = 1	No = 0
50.		Breastfeeding	Yes = 1	No = 0
51.		Keeping the baby warm	Yes = 1	No = 0
52.		Family planning	Yes = 1	No = 0

^a^Advantageous

^b^Disadvantageous groups

**Table 5:** Odds ratio estimates from the binary logistic regression model showing mother and child (0–5 years) health care by levels of women’s FHWE among the poor and non-poor women delivered in public health institutions in India, 2015–2016

Predictor variables	Antenatal care (4 or > 4 times)			Place of delivery (public institutions)			Child full immunisation (12–23 months)			Infant postnatal care (within 2 days of delivery)		
	Odds ratio (SE^a^)			Odds ratio (SE)			Odds ratio (SE)			Odds ratio (SE)		
	Poor (*n* = 51,153)	Non-poor (*n* = 54,462)	Total (*n* = 105,615)	Poor (*n* = 130,132)	Non-poor (*n* = 129,495)	Total (*n* = 259,627)	Poor (*n* = 20,520)	Non-poor (*n* = 18,783)	Total (*n* = 39,303)	Poor (*n* = 51,153)	Non-poor (*n* = 54,462)	Total (*n* = 105,615)
Panel A (unadjusted)												
Level of FHWE												
Low®												
Medium	2.124* (0.07)	1.559* (0.04)	1.699* (0.03)	1.752* (0.03)	1.393* (0.02)	1.533* (0.02)	1.549* (0.06)	1.558* (0.06)	1.553* (0.04)	1.891* (0.08)	1.676* (0.06)	1.774* (0.05)
High	6.566* (0.21)	2.326* (0.06)	3.430* (0.06)	2.594* (0.04)	2.681* (0.03)	2.342* (0.02)	2.381* (0.09)	2.158* (0.09)	2.289* (0.06)	4.337* (0.16)	3.088* (0.10)	3.593* (0.08)
Panel B (adjusted)^b^												
Level of FHWE												
Low®												
Medium	1.976* (0.07)	1.560* (0.05)	1.685* (0.04)	1.655* (0.02)	1.368* (0.02)	1.494* (0.02)	1.539* (0.06)	1.544* (0.07)	1.550* (0.04)	1.881* (0.08)	1.677* (0.06)	1.766* (0.05)
High	5.560* (0.19)	2.357* (0.07)	3.485* (0.07)	2.408* (0.04)	2.079* (0.03)	2.226* (0.02)	2.325* (0.09)	2.128* (0.09)	2.255* (0.07)	4.312* (0.16)	3.087* (0.09)	3.608* (0.09)

*FHWE* Frontline Health Worker Interaction, *CFI* child full immunisation

**p* < 0.05

® Reference group

^a^Standard error in the parentheses

^b^Models was controlled for birth order, current age of women (15–59), woman’s education, women’s partner education, woman’s occupation, woman’s partner occupation, caste, religion, place of residence, region. Detailed tables are presented in Additional file 6: Table S4

**Table 6:** Hazard ratio estimates from the Cox proportional hazard regression model: child survival outcome by mother’s level of FHWE among the poor and non-poor women delivered in public health institutions in India, 2015–2016

Predictor variables	Hazard ratio (SE^a^)		
	Poor (*n* = 71,446)	Non-poor (*n* = 69,582)	Total (*n* = 141,028)
Panel A (unadjusted)			
Level of FHWE			
Low®			
Medium	0.545* (0.03)	0.646* (0.05)	0.580* (0.03)
High	0.208* (0.02)	0.264* (0.03)	0.227* (0.01)
Panel B (adjusted)^b^			
Level of FHWE			
Low®			
Medium	0.547* (0.04)	0.625* (0.5)	0.576* (0.03)
High	0.210* (0.02)	0.248* (0.2)	0.225* (0.01)

*FHWE* Frontline Health Worker Interaction

**p* < 0.05

® Reference group

^a^Standard error

^b^Model was controlled for birth order, current age of women (15–59), woman’s education, women’s partner education, woman’s occupation, woman’s partner occupation, caste, religion, place of residence, region. Detailed tables are presented in Additional file 6: Table S5

## Abbreviations

ANM: Auxiliary Nurse Midwife
ASHA: Accredited Social Health Activist
AWW: Anganwadi worker
BLR: Binary Logistic Regression
FHWE: Frontline Health Worker Engagement
MCH: Maternal, and Child Health
NFHS: National Family Health Survey
NHM: National Health Mission
