# Supplementary figures and images for: Does engagement with frontline health workers improve maternal and child healthcare utilisation and outcomes in India?
Source: Hum Resour Health. 2021 Apr 1;19:45. doi: 10.1186/s12960-021-00592-1 (PMC8017836; doi:10.1186/s12960-021-00592-1)

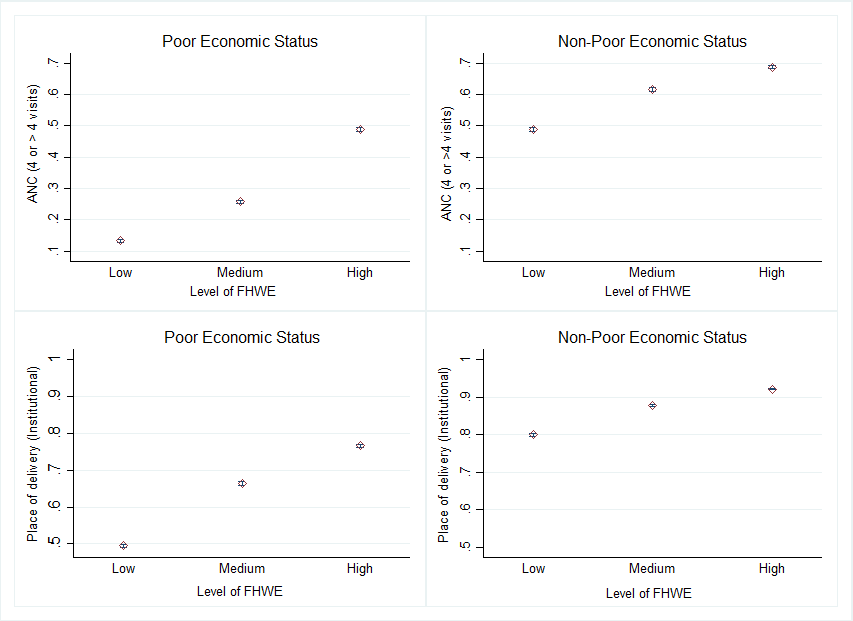

Supplement: Supplementary file 1 — Additional file 1: Fig S1. Maternal health care by FHWE for Poor and Non-Poor women in India, 2015–2016. [file 12960_2021_592_MOESM1_ESM.tif]

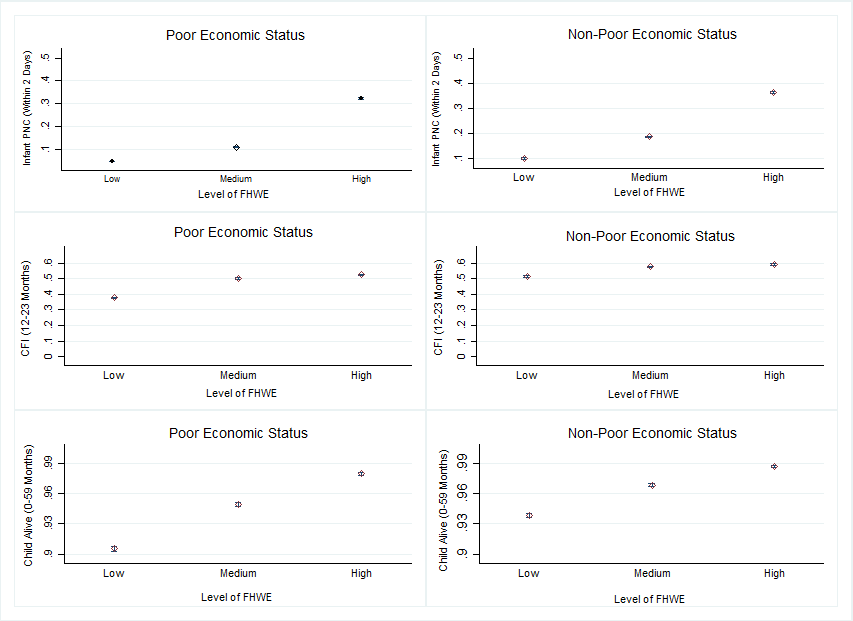

Supplement: Supplementary file 2 — Additional file 2: Fig S2. Child health care and outcomes by FHWE for Poor and Non-Poor women in India, 2015–2016. [file 12960_2021_592_MOESM2_ESM.tif]

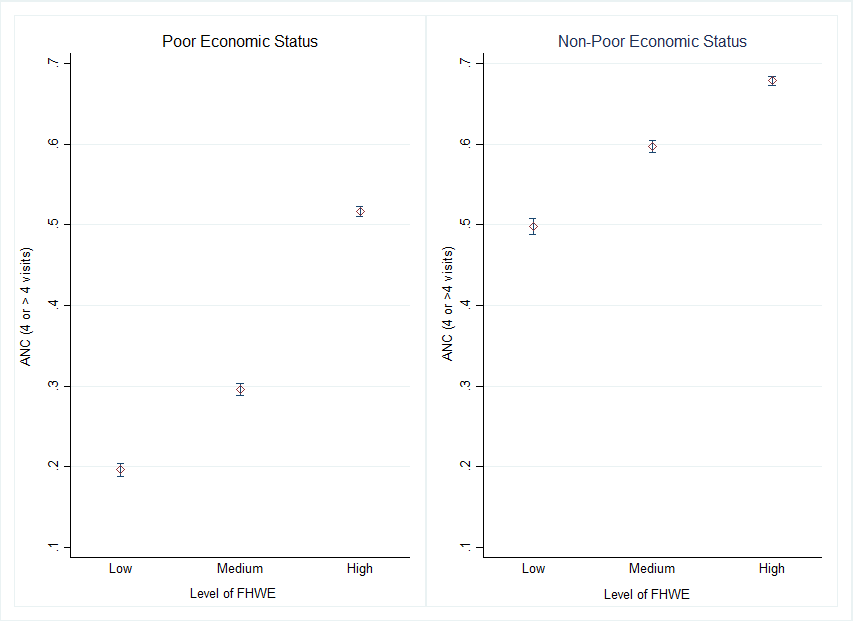

Supplement: Supplementary file 3 — Additional file 3: Fig S3. ANCs utilisation by FHWE for Poor and Non-poor women delivered in public health institutions in India, 2015–2016. [file 12960_2021_592_MOESM3_ESM.tif]

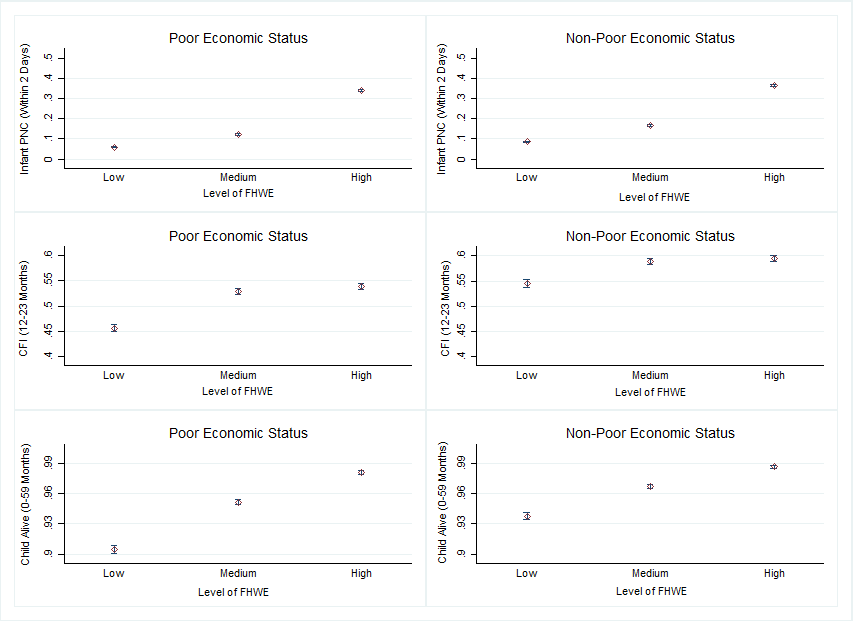

Supplement: Supplementary file 4 — Additional file 4: Fig S4. Child health care and outcomes by FHWE for Poor and Non-poor women delivered in public health institutions in India, 2015–2016. [file 12960_2021_592_MOESM4_ESM.tif]

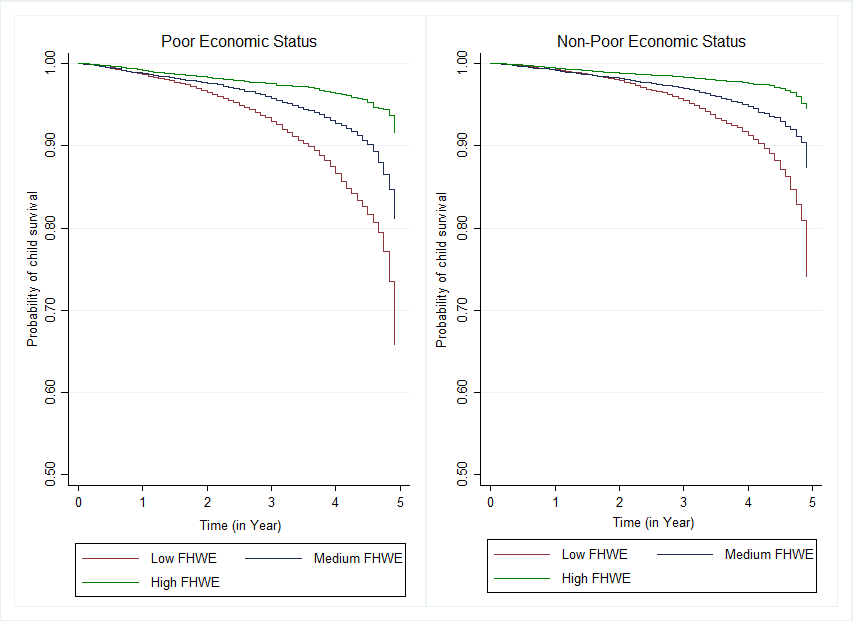

Supplement: Supplementary file 5 — Additional file 5: Fig S5. Kaplan–Meier survival estimates by level of FHWE for the Poor and Non-Poor women delivered in public health institutions in India, 2015–2016. [file 12960_2021_592_MOESM5_ESM.tif]
