# Supplementary material for: Does engagement with frontline health workers improve maternal and child healthcare utilisation and outcomes in India?
Source: Hum Resour Health. 2021 Apr 1;19:45. doi: 10.1186/s12960-021-00592-1 (PMC8017836; doi:10.1186/s12960-021-00592-1)
Supplement: Supplementary file 6 — Additional file 6: Table S1. Descriptive statistics of background characteristics by maternal and child health outcomes in India, 2015–2016. Table S2. Descriptive statistics of background characteristics by maternal and child health outcomes among women delivered in public health institutions in India, 2015–2016. Table S3. Descriptive statistics of background characteristics by maternal and child health outcomes in India, 2015–2016. Table S4. Odds ratio by using Binary Logistic Regression (BLR) model of mother and child (0–5 years) health outcomes by levels of FHWE among the poor and non-poor women delivered in public health institutions in India, 2015–2016. Table S5. Hazard ratio by using Cox Proportional Hazard regression model of mother and child (0–5 years) health outcomes by FHWE Level, among the poor and non-poor women delivered in public health institutions in India, 2015–2016. Table S6. Odds ratio by using Binary Logistic Regression (BLR) model of mother and child (0–5 years) health outcomes by levels of FHWE among the poor and non-poor of rural women in India, 2015–2016. Table S7. Hazard ratio by using Cox Proportional Hazard regression model of child survival outcome by FHWE Level, among the poor and non-poor of rural women in India, 2015–2016. [file 12960_2021_592_MOESM6_ESM.docx]

| Table A4. Odds ratio estimates from binary logistic regression model showing mother and child (0-5 years) health care by levels of women’s FHWE among the poor and non-poor **of** rural women in India, 2015-16 | | | | | | | | | | | | |
| --- | --- | --- | --- | --- | --- | --- | --- | --- | --- | --- | --- | --- |
| Predictor Variables | Antenatal Care (4 or > 4 times) | | | Place of delivery (Public Institutions) | | | Child Full Immunization (12-23 months) | | | Infant Postnatal care (within 2 days of delivery) | | |
|  | Odds Ratio (SE) | | | Odds Ratio (SE) | | | Odds Ratio (SE) | | | Odds Ratio (SE) | | |
|  | Poor  (*n*=83918) | Non-Poor (*n*=59147) | Total (*n*=143065) | Poor (*n*=120700) | Non-Poor (*n*=77548) | Total (*n*=198248) | Poor (*n*=34385) | Non-Poor (*n*=20989) | Total (*n*=55374) | Poor (*n*=83918) | Non-Poor (*n*=59147) | Total (*n*=143065) |
| **Panel A *(Unadjusted)*** | | | | | | | | | | | | |
| ***Level of FHWE*** | | | | | | | | | | | | |
| Low |  |  |  |  |  |  |  |  |  |  |  |  |
| Medium | 2.524^*^ (0.07) | 1.815^*^ (0.05) | 2.254^*^ (0.04) | 1.807^*^ (0.03) | 1.706^*^ (0.05) | 1.902^*^ (0.03) | 1.706^*^ (0.05) | 1.549^*^ (0.06) | 1.708^*^ (0.04) | 2.003^*^ (0.06) | 1.732^*^ (0.06) | 1.962^*^ (0.04) |
| High | 8.735^*^ (0.22) | 3.369^*^ (0.08) | 5.846^*^ (0.09) | 2.843^*^ (0.05) | 3.049^*^ (0.09) | 3.231^*^ (0.04) | 2.802^*^ (0.08) | 2.158^*^ (0.08) | 2.678^*^ (0.06) | 4.837^*^ (0.13) | 3.193^*^ (0.09) | 4.221^*^ (0.09) |
| **Panel B *(Adjusted)^#^*** | | | | | | | | | | | | |
| ***Level of FHWE*** | | | | | | | | | | | | |
| Low |  |  |  |  |  |  |  |  |  |  |  |  |
| Medium | 2.234^*^ (0.06) | 1.685^*^ (0.05) | 1.945^*^ (0.04) | 1.688^*^ (0.03) | 1.461^*^ (0.04) | 1.644^*^ (0.02) | 1.663^*^ (0.05) | 1.599^*^ (0.07) | 1.659^*^ (0.04) | 1.978^*^ (0.06) | 1.735^*^ (0.06) | 1.899^*^ (0.04) |
| High | 6.682^*^ (0.18) | 2.816^*^ (0.08) | 4.496^*^ (0.08) | 2.583^*^ (0.05) | 2.477^*^ (0.08) | 2.597^*^ (0.04) | 2.664^*^ (0.08) | 2.204^*^ (0.09) | 2.527^*^ (0.06) | 4.817^*^ (0.14) | 3.288^*^ (0.10) | 4.089^*^ (0.09) |
| *Note:* SE represents Standard Error in the parentheses; ® Reference group; ^*^ *p < 0.05;* FHWE: Frontline Health Worker Interaction; CFI: Child Full Immunization.  ***#*** Models was controlled for birth order, current age of women (15-59), woman’s education, women’s partner education, woman’s occupation, woman’s partner occupation, caste, religion, region. Detailed tables are presented in Table S6. | | | | | | | | | | | | |

| Table A5. Hazard ratio estimates from the Cox proportional hazard regression model: child survival outcome by mother’s level of FHWE among the poor and non-poor **of rural women** in India, 2015-16 | | | |
| --- | --- | --- | --- |
| Predictor Variables | Hazard Ratio (SE) | | |
|  | Poor (*n*=120700) | Non-Poor (*n*=77548) | Total (*n*=198248) |
| **Panel A *(Unadjusted)*** | | | |
| ***Level of FHWE*** | | | |
| Low |  |  |  |
| Medium | 0.582^*^ (0.02) | 0.579^*^ (0.02) | 0.571^*^ (0.01) |
| High | 0.333^*^ (0.01) | 0.290^*^ (0.02) | 0.305^*^ (0.01) |
| **Panel B *(Adjusted)^#^*** | | | |
| ***Level of FHWE*** | | | |
| Low |  |  |  |
| Medium | 0.556^*^ (0.02) | 0.578^*^ (0.02) | 0.565^*^ (0.01) |
| High | 0.321^*^ (0.01) | 0.297^*^ (0.02) | 0.314^*^ (0.01) |
| *Note:* SE represents Standard Error in the parentheses; ® Reference group; ^*^ *p < 0.05;* FHWE: Frontline Health Worker Interaction.  ***#*** Model was controlled for birth order, current age of women (15-59), woman’s education, women’s partner education, woman’s occupation, woman’s partner occupation, caste, religion, and region. Detailed tables are presented in Table S7. | | | |

**Supplementary Tables (Only for Review purpose)**

| Table S 1. Descriptive statistics of background characteristics by maternal and child health outcomes in India, 2015-16 | | | | | | | | | | | |
| --- | --- | --- | --- | --- | --- | --- | --- | --- | --- | --- | --- |
| Predictor Variables | n | Antenatal Care | | Place of delivery | | Child Full Immunization (12-23 months) | | Infant Postnatal care | | Child Alive (0-59 months) | |
|  |  | more visit (4 or >4 times) | | Institutional birth | | Yes | | within 0-2 days | | Yes | |
|  |  | Poor (*n*=90521) | Non-Poor (*n*=100377) | Poor (*n*=130132) | Non-Poor (*n*=129495) | Poor (*n*=36995) | Non-Poor (*n*=34427) | Poor (*n*=90521) | Non-Poor (*n*=100377) | Poor (*n*=130132) | Non-Poor (*n*=129495) |
|  |  | % (SE) | % (SE) | % (SE) | % (SE) | % (SE) | % (SE) | % (SE) | % (SE) | % (SE) | % (SE) |
| *Level of FHWE* | | | | | | | | | | | |
| Low | 86544 | 12.4 (0.23) | 53.2 (0.32) | 54.8 (0.25) | 84.5 (0.18) | 44.0 (0.45) | 57.6 (0.47) | 09.9 (0.21) | 18.9 (0.25) | 89.9 (0.15) | 94.0 (0.11) |
| Medium | 86607 | 25.7 (0.28) | 65.9 (0.26) | 68.7 (0.24) | 90.4 (0.14) | 58.4 (0.46) | 66.2 (0.43) | 17.8 (0.25) | 26.7 (0.24) | 95.1 (0.11) | 96.9 (0.08) |
| High | 86476 | 53.1 (0.26) | 72.3 (0.21) | 77.3 (0.21) | 93.6 (0.11) | 69.4 (0.41) | 74.1 (0.40) | 33.7 (0.24) | 38.8 (0.23) | 98.1 (0.07) | 98.8 (0.05) |
| *Birth Order* | | | | | | | | | | | |
| 1 | 96212 | 46.2 (0.34) | 71.3 (0.22) | 78.6 (0.21) | 94.0 (0.09) | 61.8 (0.45) | 68.7 (0.35) | 24.8 (0.29) | 30.9 (0.23) | 93.6 (0.13) | 96.9 (0.07) |
| 2 | 79670 | 40.4 (0.32) | 67.6 (0.23) | 69.0 (0.25) | 90.0 (0.14) | 57.7 (0.50) | 66.7 (0.44) | 24.5 (0.27) | 30.5 (0.23) | 95.5 (0.11) | 97.3 (0.08) |
| 3 | 41607 | 31.2 (0.36) | 57.6 (0.42) | 61.5 (0.33) | 82.8 (0.29) | 54.3 (0.65) | 60.1 (0.77) | 22.1 (0.32) | 29.4 (0.38) | 94.9 (0.15) | 96.5 (0.14) |
| 3+ | 42138 | 19.5 (0.28) | 43.4 (0.56) | 51.9 (0.31) | 73.1 (0.46) | 49.0 (0.61) | 55.8 (1.06) | 20.3 (0.28) | 27.4 (0.50) | 93.2 (0.16) | 93.9 (0.25) |
| *Current age of woman* | | | | | | | | | | | |
| 15-19 | 6699 | 47.3 (0.82) | 65.7 (0.94) | 77.9 (0.64) | 92.8 (0.47) | 62.9 (1.38) | 68.9 (1.59) | 22.8 (0.67) | 30.5 (0.91) | 92.7 (0.40) | 96.3 (0.35) |
| 20-24 | 78177 | 40.8 (0.31) | 64.5 (0.26) | 73.4 (0.23) | 90.4 (0.14) | 60.3 (0.44) | 66.1 (0.41) | 24.1 (0.27) | 30.4 (0.26) | 94.2 (0.12) | 96.6 (0.09) |
| 25-29 | 99396 | 35.0 (0.29) | 66.5 (0.23) | 67.1 (0.23) | 89.5 (0.13) | 56.8 (0.44) | 66.3 (0.40) | 23.6 (0.25) | 30.4 (0.23) | 94.8 (0.11) | 97.0 (0.07) |
| 30-34 | 49005 | 29.3 (0.38) | 67.5 (0.33) | 61.4 (0.34) | 90. 0(0.19) | 52.3 (0.68) | 65.6 (0.66) | 22.3 (0.34) | 29.2 (0.33) | 94.5 (0.16) | 97.0(0.11) |
| 35-39 | 19212 | 24.4 (0.52) | 65.0 (0.61) | 53.9 (0.53) | 88.4 (0.39) | 48.2 (1.12) | 68.2 (1.27) | 20.9 (0.49) | 31.9 (0.59) | 93.7 (0.26) | 96.1 (0.23) |
| 40-44 | 5504 | 18.4 (0.81) | 59.9 (1.41) | 47.2 (0.93) | 82.0 (1.04) | 46.4 (2.14) | 64.9 (3.47) | 18.8 (0.82) | 30.4 (1.32) | 92.1 (0.51) | 94.7 (0.61) |
| 45-49 | 1634 | 14.6 (1.22) | 44.6 (3.43) | 40.9 (1.55) | 67.4 (3.12) | 49.1 (3.73) | 52.6 (10.36) | 15.0 (1.23) | 29.3 (3.14) | 87.9 (1.03) | 89.6 (2.03) |
| *Education status of women* | | | | | | | | | | | |
| Illiterate | 81087 | 23.8 (0.21) | 45.3 (0.46) | 57.8 (0.20) | 75.0 (0.34) | 50.9 (0.39) | 55.3 (0.73) | 20.4 (0.20) | 27. 0 (0.41) | 93.8 (0.10) | 94.8 (0.17) |
| Primary | 37938 | 39.7 (0.40) | 55.6 (0.49) | 68.2 (0.32) | 81.9 (0.33) | 59.1 (0.63) | 62.9 (0.77) | 24.9 (0.37) | 29.0 (0.45) | 94.1 (0.16) | 95.4 (0.18) |
| Secondary | 116646 | 48.0 (0.31) | 68.0 (0.19) | 79.5 (0.21) | 92.0 (0.09) | 64.2 (0.46) | 67.8 (0.33) | 25.8 (0.27) | 30.7 (0.19) | 95.1 (0.11) | 97.0 (0.06) |
| Higher | 23956 | 46.3 (1.35) | 75.8 (0.29) | 88.1 (0.77) | 97.0 (0.11) | 65.3 (2.12) | 71.5 (0.58) | 25.7(1.19) | 31.5 (0.32) | 95.4 (0.50) | 98.0 (0.09) |
| *Husband/ partner's educational status* | | | | | | | | | | | |
| Illiterate | 8181 | 28.9 (0.70) | 55.3 (1.53) | 57.0 (0.63) | 78.4 (1.09) | 52.2 (1.16) | 56.8 (2.36) | 21.1 (0.63) | 28.8 (1.39) | 92.9 (0.33) | 94.2 (0.62) |
| Primary | 6587 | 37.9 (0.92) | 63.6 (1.18) | 67.1 (0.74) | 82.4 (0.80) | 57.0 (1.48) | 60.7 (2.08) | 24.8 (0.82) | 30.6 (1.13) | 94.8 (0.35) | 95.4 (0.44) |
| Secondary | 24511 | 40.7 (0.62) | 69.2 (0.44) | 74.4 (0.47) | 91.1 (0.24) | 61.7 (0.95) | 70.4 (0.72) | 24.5 (0.55) | 31.4 (0.43) | 94.9 (0.23) | 96.8 (0.15) |
| Higher | 5783 | 40.8 (2.35) | 74.8 (0.65) | 82.2 (1.57) | 95.6 (0.27) | 62.1 (3.80) | 70.6 (1.24) | 24.5 (2.06) | 29.4 (0.68) | 95.7 (0.84) | 98.0 (0.19) |
| Don't know/missing | 214565 | 34.5 (0.18) | 65.1 (0.16) | 66.6 (0.15) | 89.6 (0.09) | 56.6 (0.29) | 65.7 (0.28) | 22.9 (0.16) | 30.2 (0.16) | 94.3 (0.07) | 96.8 (0.05) |
| *Occupation of woman* | | | | | | | | | | | |
| Not working | 34426 | 36.3 (0.49) | 69.1 (0.37) | 68.7 (0.39) | 90.9 (0.21) | 57.8 (0.76) | 68.7 (0.64) | 22.4 (0.42) | 30.0 (0.37) | 94.5 (0.19) | 96.8 (0.12) |
| White collar | 1382 | 35.9 (4.01) | 74.2 (1.48) | 74.2 (3.01) | 95.5 (0.65) | 80.2 (5.30) | 73.3 (2.96) | 28.1 (3.76) | 33.2 (1.59) | 96.0 (1.35) | 98.4 (0.44) |
| Agricultural worker | 5974 | 36.0 (0.94) | 66.8 (1.38) | 65.2 (0.79) | 86.7 (0.87) | 53.4 (1.57) | 67.3 (2.35) | 25.4 (0.85) | 34.0 (1.39) | 93.9 (0.39) | 95.4 (0.15) |
| Service/manual work | 3093 | 38.9 (1.54) | 70.8 (1.33) | 62.7 (1.29) | 87.2 (0.88) | 64.7 (2.40) | 66.3 (2.49) | 28.3 (1.42) | 33.8 (1.38) | 93.2 (0.67) | 96.6 (0.19) |
| Don’t know/missing | 214752 | 34.5 (0.18) | 65.1(0.16) | 66.6 (0.15) | 89.6 (0.09) | 56.6 (0.29) | 65.7 (0.28) | 22.9 (0.16) | 30.2 (0.16) | 94.3 (0.07) | 96.8 (0.05) |
| *Husband/ partner's occupation* | | | | | | | | | | | |
| Not working | 1939 | 32.1 (1.85) | 65.2 (1.70) | 66.4 (1.57) | 91.0 (0.91) | 55.7 (3.20) | 61.0 (2.97) | 19.2 (1.56) | 28.8 (1.62) | 93.8 (0.80) | 95.4 (0.13) |
| White collar | 8945 | 34.6 (1.25) | 72.2(0.59) | 69.2 (1.01) | 92.9 (0.30) | 57.4 (2.11) | 69.9 (1.05) | 23.7 (1.12) | 29.8 (0.60) | 94.7 (0.49) | 97.6 (0.39) |
| Agricultural worker | 14614 | 38.2 (0.64) | 63.6 (0.81) | 66.7 (0.52) | 89.1 (0.46) | 55.0 (1.01) | 64.3 (1.33) | 22.6 (0.55) | 29.3 (0.77) | 94.5 (0.25) | 96.1 (0.54) |
| Service/manual work | 19161 | 36.2 (0.63) | 69.9 (0.51) | 68.5 (0.51) | 89.5 (0.30) | 60.9 (0.98) | 70.7 (0.86) | 25.0 (0.57) | 32.2 (0.52) | 94.3 (0.26) | 96.7 (0.48) |
| Don’t know | 214968 | 34.5 (0.18) | 65.1 (0.16) | 66.6 (0.15) | 89.6 (0.09) | 56.6 (0.29) | 65.7 (0.28) | 22.9 (0.16) | 30.2 (0.16) | 94.3 (0.07) | 96.8 (0.05) |
| *Caste* | | | | | | | | | | | |
| Others | 45019 | 43.2 (0.49) | 69.0 (0.28) | 65.8 (0.40) | 91.2(0.15) | 57.4 (0.78) | 64.7 (0.51) | 22.1 (0.42) | 29.8 (0.28) | 95.0 (0.18) | 97.1 (0.67) |
| SC | 49051 | 35.3 (0.32) | 65.6 (0.35) | 69.9 (0.26) | 89.3(0.20) | 59.4 (0.53) | 68.6 (0.59) | 23.8 (0.29) | 32.3 (0.35) | 93.9 (0.14) | 96.6 (0.18) |
| ST | 52199 | 40.2 (0.42) | 64.4 (0.66) | 62.1 (0.34) | 85.0 (0.43) | 52.9 (0.67) | 64.2 (1.15) | 26.1 (0.37) | 28.2 (0.62) | 94.4 (0.16) | 97.0 (0.28) |
| OBC | 101,786 | 28.5 (0.25) | 63.6 (0.22) | 67.7 (0.21) | 89.5 (0.12) | 56.4 (0.41) | 66.2 (0.37) | 21.9 (0.23) | 30.5 (0.21) | 94.1 (0.10) | 96.5 (0.18) |
| Don’t know | 11572 | 46.9 (0.80) | 71.9 (0.66) | 60.4 (0.68) | 91.7 (0.36) | 58.2 (1.32) | 67.6 (1.19) | 20.4 (0.65) | 25.5 (0.64) | 95.7 (0.28) | 97.3 (0.05) |
| *Religion* | | | | | | | | | | | |
| Hindus | 187573 | 34.7 (0.19) | 65.8 (0.17) | 69.9 (0.15) | 91.0 (0.09) | 57.8 (0.30) | 67.4 (0.29) | 23.5 (0.17) | 30.0 (0.16) | 94.2 (0.07) | 96.8 (0.06) |
| Muslims | 40950 | 32.5 (0.41) | 63.2 (0.37) | 52.7 (0.36) | 83.2 (0.25) | 51.1 (0.66) | 58.8 (0.62) | 20.0 (0.35) | 29.5 (0.35) | 94.6 (0.16) | 96.5 (0.12) |
| Christians | 20934 | 44.7 (1.36) | 73.4 (0.87) | 55.4 (1.15) | 92.0 (0.48) | 56.1 (2.24) | 68.8 (1.56) | 22.8 (1.15) | 29.5 (0.90) | 95.9 (0.46) | 98.0 (0.25) |
| Others | 10170 | 52.9 (1.27) | 72.7 (0.72) | 60.5 (1.06) | 93.2 (0.37) | 65.6 (1.91) | 78.0 (1.21) | 27.9 (1.14) | 39.4 (0.79) | 95.1 (0.46) | 97.3 (0.23) |
| *Place of residence* | | | | | | | | | | | |
| Rural | 198248 | 33.9 (0.17) | 61.8 (0.21) | 66.4 (0.14) | 88.3 (0.12) | 56.8 (0.28) | 67.9 (0.34) | 22.9 (0.15) | 31.2 (0.19) | 94.2 (0.07) | 96.4 (0.07) |
| Urban | 61379 | 45.7 (0.62) | 70.4 (0.21) | 71.2 (0.46) | 91.4 (0.11) | 56.5 (0.94) | 64.1 (0.38) | 23.9 (0.53) | 29.2 (0.20) | 95.4 (0.21) | 97.2 (0.07) |
| *Region* | | | | | | | | | | | |
| Northern | 48703 | 30.2 (0.59) | 57.6 (0.36) | 72.2 (0.47) | 87.3 (0.22) | 49.3 (0.99) | 69.5 (0.58) | 20.9 (0.53) | 30.1 (0.34) | 94.6 (0.24) | 96.6 (0.12) |
| Central | 75645 | 21.3 (0.25) | 46.3 (0.34) | 64.2 (0.24) | 81.0 (0.23) | 49.8 (0.47) | 62.4 (0.55) | 22.9 (0.26) | 32.9 (0.32) | 92.7 (0.13) | 94.8 (0.13) |
| Eastern | 54075 | 33.5 (0.26) | 62.2 (0.40) | 62.6 (0.22) | 87.1 (0.25) | 65.3 (0.41) | 76.2 (0.63) | 21.5 (0.23) | 28.1(0.38) | 94.9 (0.10 | 97.2 (0.13) |
| North-eastern | 37167 | 40.9 (0.74) | 65.4 (0.91) | 58.1 (0.65) | 85.8 (0.62) | 47.2 (1.29) | 62.3 (1.76) | 22.4 (0.63) | 23.7 (0.81) | 94.7 (0.29) | 97.2 (0.29) |
| Western | 18276 | 59.6 (0.61) | 77.1 (0.31) | 77.9 (0.43) | 94.5 (0.15) | 48.9 (0.94) | 56.8 (0.65) | 27.6 (0.56) | 27.2 (0.33) | 95.9 (0.20) | 97.5 (0.10) |
| Southern | 25761 | 72.3 (0.56) | 80.7 (0.23) | 88.2 (0.33) | 97.0 (0.09) | 64.5(0.95) | 68.4 (0.47) | 29.1 (0.57) | 32.2 (0.27) | 95.7 (0.21) | 97.7 (0.08) |
| Total | 259627 | 34.8 (0.17) | 65.8 (0.15) | 66.8 (0.14) | 89.8 (0.08) | 56.8 (0.27) | 66.2 (0.25) | 23.1 (0.15) | 30.3 (0.14) | 94.3 (0.07) | 96.8 (0.05) |
| Note: SE represents Standard Error | | | | | | | | | | | |

| Table S 2. Descriptive statistics of background characteristics by maternal and child health outcomes among women delivered in public health institutions in India, 2015-2016 | | | | | | | | | | | |
| --- | --- | --- | --- | --- | --- | --- | --- | --- | --- | --- | --- |
| Predictor Variables | n | Antenatal care | | Place of delivery | | Child full immunization (12-23 months) | | Infant Postnatal Care | | Child alive (0-59 months) | |
|  |  | more visit (4 or >4 times) | | Public institutional delivery | | Yes | | within 0-2 days | | Yes | |
|  |  | Poor (*n*=51153) | Non-Poor (*n*=54462) | Poor (*n*=130132) | Non-Poor (*n*=129495) | Poor (*n*=20520) | Non-Poor (*n*=18783) | Poor (*n*=51153) | Non-Poor (*n*=54462) | Poor (*n*=71446) | Non-Poor (*n*=69582) |
|  |  | % (SE) | % (SE) | % (SE) | % (SE) | % (SE) | % (SE) | % (SE) | % (SE) | % (SE) | % (SE) |
| *Level of FHWE* | | | | | | | | | | | |
| Low | 36199 | 15.8 (0.39) | 50.3 (0.53) | 43.5 (0.25) | 38.9 (0.25) | 50.6 (0.68) | 57.3 (0.74) | 11.4 (0.34) | 17.2 (0.40) | 90.2 (0.22) | 93.4 (0.20) |
| Medium | 47620 | 28.5 (0.39) | 61.2 (0.40) | 57.4 (0.25) | 47.1 (0.24) | 61.4 (0.60) | 67.7 (0.62) | 19.5 (0.34) | 25.8 (0.36) | 95.3 (0.14) | 96.8 (0.12) |
| High | 57209 | 55.2 (0.32) | 70.2 (0.28) | 66.7 (0.24) | 58.6 (0.23) | 70.9 (0.53) | 74.3 (0.52) | 35.8 (0.30) | 39.0 (0.30) | 98.2 (0.08) | 98.8 (0.07) |
| *Birth Order* | | | | | | | | | | | |
| 1 | 55990 | 49.2 (0.42) | 68.2 (0.34) | 63.5 (0.25) | 47.9 (0.20) | 65.5 (0.55) | 70.7 (0.49) | 26.5 (0.37) | 31.5 (0.33) | 94.4 (0.15) | 96.7 (0.11) |
| 2 | 44600 | 45.4 (0.41) | 65.9 (0.34) | 57.9 (0.27) | 49.9 (0.23) | 62.8 (0.64) | 68.3 (0.61) | 27.9 (0.37) | 31.8 (0.33) | 95.9 (0.14) | 97.3 (0.11) |
| 3 | 21754 | 36.1 (0.50) | 58.0 (0.58) | 52.9 (0.34) | 49.9 (0.39) | 59.2 (0.87) | 60.2 (1.09) | 25.9 (0.46) | 30.7 (0.54) | 95.8 (0.19) | 97.1 (0.19) |
| 3+ | 18684 | 23.5 (0.44) | 45.2 (0.81) | 44.5 (0.31) | 47.4 (0.52) | 55.4 (0.89) | 56.1 (1.49) | 25.6 (0.45) | 29.1 (0.74) | 94.3 (0.22) | 94.9 (0.33) |
| *Current age of Women* | | | | | | | | | | | |
| 15-19 | 4155 | 51.0 (1.02) | 64.3 (1.26) | 63.9 (0.74) | 55.6 (0.92) | 65.7 (1.69) | 68.8 (2.15) | 23.9 (0.87) | 30.9 (1.21) | 93.9 (0.46) | 96.6 (0.45) |
| 20-24 | 46061 | 44.7 (0.39) | 63.8 (0.37) | 60.6 (0.25) | 53.3 (0.24) | 64.7 (0.55) | 67.9 (0.55) | 26.8 (0.34) | 31.8 (0.35) | 94.9 (0.15) | 96.9 (0.12) |
| 25-29 | 54319 | 39.9 (0.39) | 64.3 (0.34) | 56.1 (0.24) | 48.4 (0.21) | 61.5 (0.57) | 68.4 (0.56) | 27.0 (0.35) | 31.4 (0.33) | 95.4 (0.14) | 96.9 (0.11) |
| 30-34 | 24734 | 35.2 (0.54) | 65.3 (0.52) | 52.1 (0.35) | 43.8 (0.32) | 57.3 (0.93) | 64.4 (0.99) | 26.8 (0.49) | 29.5 (0.49) | 95.4 (0.20) | 96.8 (0.17) |
| 35-39 | 8938 | 30.9 (0.81) | 61.9 (0.96) | 45.1 (0.53) | 41.8 (0.59) | 57.7 (1.67) | 69.6 (2.02) | 26.4 (0.78) | 33.7 (0.93) | 94.8 (0.35) | 96.5 (0.34) |
| 40-44 | 2242 | 26.1 (1.44) | 61.3 (2.35) | 39.8 (0.91) | 34.3 (1.28) | 59.6 (3.37) | 66.8 (5.67) | 25.6 (1.43) | 34.5 (2.29) | 92.4 (0.78) | 95.0 (1.01) |
| 45-49 | 579 | 23.9 (2.41) | 43.6 (6.28) | 36.9 (1.53) | 31.5 (3.09) | 50.1 (5.70) | 36.0 (2.54) | 24.3 (2.42) | 31.8 (5.89) | 92.8 (1.34) | 93.8 (2.88) |
| *Education status of Women* | | | | | | | | | | | |
| Illiterate | 40278 | 29.1 (0.32) | 49.3 (0.65) | 49.2 (0.21) | 50.6 (0.39) | 57.6 (0.54) | 58.5 (1.01) | 24.7 (0.31) | 27.2 (0.57) | 94.7 (0.13) | 95.7 (0.22) |
| Primary | 21176 | 43.5 (0.53) | 56.2 (0.66) | 57.2 (0.34) | 54.8 (0.42) | 62.5 (0.81) | 65.9 (1.02) | 28.3 (0.48) | 30.0 (0.61) | 94.7 (0.20) | 96.2 (0.21) |
| Secondary | 69756 | 51.4 (0.37) | 67.1 (0.26) | 65.1 (0.25) | 52.4 (0.18) | 66.8 (0.56) | 69.2 (0.44) | 28.1 (0.34) | 32.2 (0.26) | 95.7 (0.13) | 97.1 (0.08) |
| Higher | 9818 | 41.2 (1.69) | 69.8 (0.56) | 62.3 (1.15) | 32.7 (0.30) | 68.1 (2.62) | 73.2 (0.99) | 24.5 (1.48) | 31.9 (0.57) | 95.9 (0.59) | 97.5 (0.17) |
| *Husband/ partner's educational status* | | | | | | | | | | | |
| Illiterate | 3985 | 37.3 (1.05) | 58.1 (2.14) | 48.9 (0.63) | 50.4 (1.33) | 57.0 (1.60) | 63.5 (3.41) | 25.7 (1.95) | 28.4 (1.95) | 94.0 (0.43) | 94.4 (0.85) |
| Primary | 3674 | 43.9 (1.22) | 64.7 (1.59) | 57.5 (0.78) | 52.9 (1.04) | 65.7 (1.87) | 69.9 (2.75) | 28.7 (1.11) | 28.3 (1.50) | 95.1 (0.45) | 94.7 (0.65) |
| Secondary | 14253 | 45.9 (0.80) | 68.6 (0.61) | 60.8 (0.52) | 50.4 (0.42) | 65.3 (1.19) | 72.1 (0.98) | 27.7 (0.72) | 32.9 (0.62) | 95.9 (0.27) | 97.0 (0.19) |
| Higher | 2545 | 44.1 (3.00) | 67.9 (1.21) | 61.9 (1.99) | 33.1 (0.64) | 71.7 (4.69) | 73.5 (2.10) | 24.2 (2.58) | 29.0 (1.18) | 96.8 (0.92) | 98.0 (0.33) |
| Don't Know/missing | 116571 | 39.8 (0.25) | 63.4 (0.24) | 55.6 (0.15) | 49.3 (0.15) | 61.8 (0.38) | 66.9 (0.39) | 26.5 (0.22) | 31.3 (0.23) | 95.0 (0.09) | 96.9 (0.08) |
| *Occupation of woman* | | | | | | | | | | | |
| Not working | 18750 | 42.5 (0.65) | 67.7 (0.55) | 57.2 (0.42) | 46.6 (0.36) | 64.1 (0.97) | 71.7 (0.89) | 25.5 (0.57) | 30.7 (0.55) | 95.7 (0.22) | 96.8 (0.18) |
| White collar | 678 | 46.1 (5.30) | 62.4 (2.70) | 62.4 (3.33) | 37.3 (1.54) | 83.9 (5.61) | 74.3 (5.16) | 32.1 (0.49) | 29.4 (2.54) | 95.4 (1.83) | 97.4 (0.82) |
| Agricultural worker | 3185 | 42.6 (1.31) | 63.9 (1.95) | 53.5 (0.82) | 51.5 (1.28) | 55.2 (2.14) | 65.1 (3.30) | 30.0 (1.22) | 33.8 (1.91) | 94.1 (0.53) | 95.9 (0.71) |
| Service/manual work | 1742 | 50.8 (2.11) | 71.4 (1.86) | 54.5 (1.33) | 50.5 (1.31) | 68.3 (3.08) | 74.9 (3.17) | 36.4 (2.03) | 37.5 (1.99) | 93.7 (0.88) | 96.1 (0.72) |
| Don’t know/missing | 116673 | 39.8 (0.25) | 63.4 (0.24) | 55.6 (0.16) | 49.3 (0.15) | 61.8 (0.38) | 66.9 (0.39) | 26.6 (0.22) | 31.3 (0.22) | 95.0 (0.09) | 96.9 (0.08) |
| *Husband/ partner's occupation* | | | | | | | | | | | |
| Not working | 1109 | 32.6 (2.55) | 64.7 (2.36) | 52.9 (1.66) | 54.3 (1.59) | 60.7 (4.15) | 62.8 (3.76) | 27.4 (2.42) | 28.6 (2.22) | 94.7 (1.03) | 95.5 (0.89) |
| White collar | 4345 | 40.9 (1.72) | 68.1 (0.99) | 55.5 (1.09) | 38.4 (0.57) | 62.9 (2.75) | 70.3 (1.67) | 26.5 (1.54) | 28.7 (0.96) | 95.9 (0.58) | 97.5 (0.29) |
| Agricultural worker | 7992 | 44.9 (0.87) | 62.0 (1.11) | 56.3 (0.55) | 53.3 (0.73) | 60.6 (1.32) | 68.7 (1.77) | 26.4 (0.77) | 29.2 (1.04) | 95.3 (0.31) | 96.3 (0.38) |
| Service/manual work | 10803 | 43.1 (0.86) | 69.8 (0.73) | 57.2 (0.54) | 48.8 (0.49) | 66.3 (1.24) | 74.7 (1.67) | 28.5 (0.77) | 34.4 (0.76) | 95.2 (0.31) | 96.8 (0.25) |
| Don’t know/missing | 116779 | 39.8 (0.25) | 63.4 (0.24) | 55.6 (0.16) | 49.3 (0.15) | 61.8 (0.38) | 66.9 (0.39) | 26.5 (0.22) | 31.3 (0.23) | 95.0 (0.09) | 96.9 (0.01) |
| *Caste* | | | | | | | | | | | |
| Others | 22160 | 47.9 (0.69) | 64.9 (0.45) | 51.9 (0.42) | 41.5 (0.27) | 62.7 (1.04) | 65.7 (0.79) | 25.2 (0.59) | 29.8 (0.43) | 95.1 (0.25) | 97.2 (0.14) |
| SC | 29879 | 41.5 (0.43) | 65.8 (0.45) | 59.7 (0.28) | 60.1 (0.32) | 63.4 (0.66) | 70.0 (0.74) | 27.4 (0.39) | 33.4 (0.45) | 94.8 (0.16) | 96.7 (0.15) |
| ST | 27546 | 48.2 (0.56) | 65.7 (0.86) | 55.8 (0.36) | 56.4 (0.60) | 59.5 (0.88) | 65.6 (1.50) | 31.7 (0.53) | 29.7 (0.83) | 95.2 (0.20) | 97.3 (0.26) |
| OBC | 54436 | 32.3 (0.34) | 61.8 (0.33) | 54.5 (0.22) | 47.0 (0.20) | 61.8 (0.54) | 67.8 (0.53) | 24.5 (0.31) | 31.9 (0.31) | 95.0 (0.13) | 96.6 (0.11) |
| Don’t know/missing | 7007 | 55.9 (1.06) | 70.8 (0.88) | 53.8 (0.69) | 55.9 (0.65) | 62.9 (1.77) | 69.2 (1.51) | 26.6 (0.94) | 26.6 (0.86) | 96.6 (0.34) | 97.3 (0.29) |
| *Religion* | | | | | | | | | | | |
| Hindus | 106876 | 39.7 (0.25) | 63.5 (0.24) | 58.5 (0.16) | 49.8 (0.16) | 62.5 (0.38) | 68.5 (0.40) | 26.9 (0.22) | 31.2 (0.23) | 95.1 (0.09) | 96.8 (0.08) |
| Muslims | 19441 | 40.1 (0.64) | 63.9 (0.55) | 43.9 (0.36) | 43.9 (0.33) | 58.1 (0.97) | 61.6 (0.92) | 24.5 (0.56) | 29.5 (0.53) | 94.7 (0.25) | 96.8 (0.18) |
| Christians | 9279 | 56.2 (2.02) | 73.0 (1.31) | 41.8 (1.14) | 44.0 (0.87) | 64.7 (3.55) | 70.2 (2.33) | 28.8 (1.84) | 31.5 (1.37) | 95.7 (0.72) | 97.5 (0.41) |
| Others | 5432 | 60.5 (1.75) | 70.7 (1.01) | 49.7 (1.11) | 53.8 (0.73) | 70.1 (2.49) | 77.5 (1.61) | 31.3 (1.66) | 40.9 (1.09) | 96.6 (0.55) | 97.1 (0.33) |
| *Place of residence* | | | | | | | | | | | |
| Rural | 110259 | 39.2 (0.24) | 60.7 (0.29) | 55.4 (0.15) | 52.8 (0.19) | 61.9 (0.37) | 69.9 (0.45) | 26.7 (0.21) | 32.4 (0.28) | 95.0 (0.08) | 96.6 (0.09) |
| Urban | 30769 | 52.8 (0.79) | 68.7 (0.32) | 59.3 (0.50) | 44.1 (0.20) | 62.5 (1.18) | 64.7 (0.56) | 26.2 (0.69) | 29.9 (0.31) | 95.5 (0.27) | 97.1 (0.10) |
| *Region* |  |  |  |  |  |  |  |  |  |  |  |
| Northern | 29692 | 35.3 (0.77) | 58.2 (0.48) | 62.4 (0.51) | 57.8 (0.32) | 56.5 (1.24) | 71.8 (0.73) | 24.6 (0.69) | 29.9 (0.44) | 94.9 (0.29) | 96.7 (0.15) |
| Central | 40356 | 25.0 (0.36) | 43.2 (0.49) | 55.1 (0.25) | 47.3 (0.29) | 54.6 (0.63) | 63.0 (0.79) | 25.9 (0.36) | 31.2 (0.46) | 93.7 (0.17) | 95.4 (0.18) |
| Eastern | 29373 | 39.2 (0.35) | 62.1 (0.56) | 53.4 (0.23) | 51.6 (0.38) | 70.9 (0.53) | 78.4 (0.84) | 25.6 (0.33) | 32.3 (0.54) | 95.8 (0.13) | 97.5 (0.16) |
| North-eastern | 18861 | 50.9 (0.99) | 66.8 (1.14) | 54.1 (0.66) | 60.9 (0.87) | 54.9 (1.75) | 62.7 (2.25) | 29.4 (0.91) | 24.9 (1.05) | 95.0 (0.39) | 97.4 (0.36) |
| Western | 8061 | 64.7 (0.81) | 74.4 (0.52) | 53.1 (0.52) | 39.6 (0.33) | 55.3 (1.26) | 56.2 (1.03) | 29.3 (0.77) | 29.4 (0.55) | 96.6 (0.26) | 97.4 (0.17) |
| Southern | 14685 | 75.0 (0.65) | 80.0 (0.35) | 67.6 (0.49) | 47.5 (0.26) | 65.6 (1.13) | 68.9 (0.68) | 31.6 (0.69) | 33.9 (0.41) | 96.2 (0.25) | 97.4 (0.12) |
| Total | 141028 | 40.3 (0.23) | 64.1 (0.21) | 55.7 (0.14) | 48.8 (0.14) | 62.0 (0.35) | 67.7 (0.36) | 26.7 (0.20) | 31.3 (0.21) | 95.1 (0.08) | 96.8 (0.07) |
| Note: SE represents Standard Error in the parentheses | | | | | | | | | | | |

| Table S 3. Descriptive statistics of background characteristics by maternal and child health outcomes in India, 2015-16 | | | | | | | | | | | |
| --- | --- | --- | --- | --- | --- | --- | --- | --- | --- | --- | --- |
| Predictor Variables | n | Antenatal Care | | Place of Delivery | | Child Full Immunization (12-23 months) | | Infant Postnatal care | | Child Alive (0-59 months) | |
|  |  | more visit (4 or >4 times) | | Institutional delivery | | Yes | | within 0-2 days | | Yes | |
|  |  | Total (*n*=190898) | Public^@^ (*n*=105615) | Total (*n*=259627) | Public^@^ (*n*=259627) | Total (*n*=71422) | Public^@^ (*n*=39303) | Total (*n*=190898) | Public^@^ (*n*=105615) | Total (*n*=259627) | Public^@^ (*n*=141028) |
|  |  | % (SE) | % (SE) | % (SE) | % (SE) | % (SE) | % (SE) | % (SE) | % (SE) | % (SE) | % (SE) |
| ***Level of FHWE*** | | | | | | | | | | | |
| Low | 86544 | 34.18 (0.22) | 33.12 (0.36) | 69.43 (0.16) | 41.28 (0.17) | 50.50 (0.33) | 53.67 (0.50) | 14.72 (0.17) | 14.29 (0.26) | 91.90 (0.09) | 91.65 (0.15) |
| Medium | 86607 | 49.07 (0.21) | 45.69 (0.29) | 80.36 (0.14) | 51.83 (0.17) | 62.40 (0.32) | 64.28 (0.43) | 22.92 (0.18) | 22.82 (0.25) | 96.10 (0.07) | 96.04 (0.09) |
| High | 86476 | 63.68 (0.17) | 62.95 (0.21) | 86.18 (0.12) | 62.27 (0.16) | 71.88 (0.29) | 72.62 (0.37) | 36.53 (0.17) | 37.46 (0.21) | 98.49 (0.04) | 98.50 (0.05) |
| ***Birth Order*** | | | | | | | | | | | |
| 1 | 96212 | 62.64 (0.19) | 60.19 (0.27) | 88.14 (0.10) | 53.83 (0.16) | 65.88 (0.28) | 68.25 (0.37) | 28.83 (0.18) | 29.41 (0.25) | 95.63 (0.07) | 95.70 (0.09) |
| 2 | 79670 | 57.39 (0.19) | 57.29 (0.27) | 81.19 (0.14) | 53.27 (0.18) | 62.56 (0.33) | 65.54 (0.44) | 28.26 (0.18) | 30.18 (0.25) | 96.52 (0.06) | 96.64 (0.09) |
| 3 | 41607 | 43.44 (0.28) | 45.77 (0.39) | 70.69 (0.23) | 51.55 (0.26) | 56.68 (0.49) | 59.56 (0.68) | 25.49 (0.25) | 28.07 (0.35) | 95.62 (0.10) | 96.33 (0.13) |
| 3+ | 42138 | 26.08 (0.26) | 29.66 (0.39) | 57.37 (0.26) | 45.24 (0.26) | 50.69 (0.53) | 55.580 (0.76) | 22.25 (0.25) | 26.57 (0.38) | 93.37 (0.13) | 94.46 (0.18) |
| ***Current age of woman*** | | | | | | | | | | | |
| 15-19 | 6699 | 54.84 (0.63) | 56.06 (0.79) | 83.97 (0.43) | 60.55 (0.58) | 65.33 (1.04) | 66.85 (1.33) | 25.96 (0.55) | 26.56 (0.71) | 94.18 (0.28) | 94.96 (0.33) |
| 20-24 | 78177 | 53.86 (0.21) | 54.51 (0.27) | 82.39 (0.13) | 56.70 (0.17) | 63.29 (0.30) | 66.28 (0.39) | 27.59 (0.19) | 29.36 (0.25) | 95.48 (0.73) | 95.89 (0.09) |
| 25-29 | 99396 | 53.79 (0.19) | 53.45 (0.26) | 79.63 (0.13) | 51.81 (0.16) | 61.75 (0.29) | 64.88 (0.40) | 27.67 (0.17) | 29.45 (0.24) | 96.02 (0.06) | 96.21 (0.09) |
| 30-34 | 49005 | 50.93 (0.27) | 50.69 (0.39) | 76.48 (0.20) | 47.72 (0.24) | 58.83 (0.48) | 60.51 (0.68) | 26.23 (0.24) | 28.19 (0.35) | 95.83 (0.09) | 96.08 (0.13) |
| 35-39 | 19212 | 43.61 (0.44) | 44.68 (0.66) | 68.91 (0.37) | 43.69 (0.39) | 56.31 (0.86) | 62.11 (1.29) | 26.10 (0.39) | 29.65 (0.60) | 94.74 (0.18) | 95.51 (0.25) |
| 40-44 | 5504 | 32.89 (0.79) | 37.25 (1.32) | 58.39 (0.76) | 38.01 (0.74) | 51.21 (1.85) | 61.39 (2.90) | 22.89 (0.71) | 28.41 (1.23) | 92.93 (0.39) | 93.17 (0.63) |
| 45-49 | 1634 | 20.62 (1.25) | 27.26 (2.29) | 45.77 (1.42) | 35.93 (1.37) | 49.52 (3.49) | 48.99 (5.48) | 17.92 (1.19) | 25.58 (2.25) | 88.27 (0.91) | 92.96 (1.22) |
| ***Education status of women*** | | | | | | | | | | | |
| Illiterate | 81087 | 28.63 (0.20) | 33.80 (0.29) | 61.54 (0.18) | 49.51 (0.18) | 51.91 (0.34) | 57.81 (0.47) | 21.88 (0.18) | 25.29 (0.27) | 94.01 (0.09) | 94.89 (0.11) |
| Primary | 37938 | 46.09 (0.32) | 48.52 (0.42) | 73.55 (0.24) | 56.25 (0.26) | 60.58 (0.49) | 63.81 (0.64) | 26.55 (0.28) | 28.99 (0.38) | 94.64 (0.12) | 95.25 (0.15) |
| Secondary | 116646 | 61.91 (0.16) | 61.53 (0.22) | 87.98 (0.10) | 56.51 (0.15) | 66.53 (0.27) | 68.23 (0.35) | 29.23 (0.15) | 30.78 (0.21) | 96.41 (0.06) | 96.58 (0.07) |
| Higher | 23956 | 73.98 (0.29) | 66.61 (0.54) | 96.41 (0.11) | 34.69 (0.29) | 71.06 (0.56) | 72.47 (0.93) | 31.16 (0.31) | 31.06 (0.53) | 97.85 (0.09) | 97.30 (0.17) |
| ***Husband/ partner's educational status*** | | | | | | | | | | | |
| Illiterate | 8181 | 34.21 (0.65) | 41.48 (0.96) | 60.97 (0.56) | 49.18 (0.57) | 53.04 (1.04) | 58.16 (1.45) | 22.61 (0.58) | 26.22 (0.86) | 93.13 (0.29) | 94.12 (0.38) |
| Primary | 6587 | 47.49 (0.75) | 51.20 (0.99) | 72.62 (0.56) | 55.87 (0.63) | 58.27 (1.21) | 66.98 (1.55) | 26.94 (0.66) | 28.55 (0.89) | 95.04 (0.27) | 94.97 (0.37) |
| Secondary | 24511 | 59.06 (0.37) | 59.41 (0.50) | 84.76 (0.24) | 54.33 (0.33) | 66.94 (0.58) | 69.19 (0.76) | 28.92 (0.34) | 30.81 (0.47) | 96.13 (0.13) | 96.58 (0.16) |
| Higher | 5783 | 71.74 (0.64) | 64.13 (1.14) | 94.31 (0.30) | 35.96 (0.62) | 69.63 (1.18) | 73.16 (1.92) | 28.99 (0.65) | 28.28 (1.07) | 97.73 (0.19) | 97.79 (0.32) |
| Don't know/missing | 214565 | 51.35 (0.13) | 51.96 (0.18) | 78.63 (0.09) | 52.29 (0.11) | 61.18 (0.20) | 64.22 (0.27) | 26.95 (0.11) | 28.99 (0.16) | 95.58 (0.05) | 95.94 (0.06) |
| ***Occupation of woman*** | | | | | | | | | | | |
| Not working | 34426 | 56.21 (0.31) | 56.38 (0.44) | 81.54 (0.21) | 51.05 (0.27) | 63.87 (0.49) | 67.98 (0.66) | 26.98 (0.28) | 28.36 (0.39) | 95.87 (0.11) | 96.32 (0.14) |
| White collar | 1382 | 68.76 (1.45) | 58.85 (2.43) | 91.76 (0.79) | 41.74 (1.42) | 74.69 (2.59) | 77.96 (3.86) | 32.52 (1.47) | 29.97 (2.26) | 97.98 (0.41) | 96.89 (0.77) |
| Agricultural worker | 5974 | 45.73 (0.81) | 49.05 (1.11) | 71.49 (0.63) | 52.88 (0.69) | 57.29 (1.32) | 57.98 (1.81) | 28.07 (0.73) | 31.17 (1.03) | 94.39 (0.32) | 94.64 (0.43) |
| Service/manual work | 3093 | 56.07 (1.06) | 61.35 (1.44) | 75.22 (0.81) | 52.43 (0.94) | 65.49 (1.73) | 71.28 (2.22) | 31.28 (0.99) | 36.98 (1.42) | 94.93 (0.41) | 94.85 (0.57) |
| Don’t know/missing | 214752 | 51.34 (0.13) | 51.95 (0.18) | 78.61 (0.09) | 52.29 (0.11) | 61.18 (0.20) | 64.22 (0.27) | 26.96 (0.11) | 29.02 (0.16) | 95.57 (0.05) | 95.94 (0.06) |
| ***Husband/ partner's occupation*** | | | | | | | | | | | |
| Not working | 1939 | 50.43 (1.33) | 50.15 (1.82) | 79.19 (0.93) | 53.60 (1.15) | 58.49 (2.18) | 61.85 (2.78) | 24.49 (1.14) | 28.07 (1.64) | 94.63 (0.52) | 95.13 (0.07) |
| White collar | 8945 | 64.77 (0.56) | 60.78 (0.88) | 87.59 (0.34) | 42.18 (0.51) | 67.04 (0.95) | 68.10 (1.43) | 28.57 (0.53) | 28.12 (0.81) | 96.96 (0.18) | 97.01 (0.27) |
| Agricultural worker | 14614 | 47.88 (0.52) | 51.17 (0.69) | 74.86 (0.38) | 55.22 (0.44) | 58.25 (0.81) | 63.32 (1.07) | 25.16 (0.45) | 27.43 (0.62) | 95.09 (0.19) | 95.68 (0.24) |
| Service/manual work | 19161 | 55.89 (0.42) | 57.63 (0.58) | 80.23 (0.29) | 52.51 (0.36) | 66.07 (0.65) | 70.35 (0.86) | 29.24 (0.39) | 31.67 (0.54) | 95.59 (0.15) | 96.03 (0.19) |
| Don’t know | 214968 | 51.35 (0.13) | 51.96 (0.18) | 78.62 (0.90) | 52.28 (0.11) | 61.17 (0.20) | 64.21 (0.27) | 26.94 (0.11) | 28.99 (0.16) | 95.57 (0.05) | 95.93 (0.06) |
| ***Caste*** | | | | | | | | | | | |
| Others | 45019 | 62.11 (0.25) | 59.52 (0.38) | 83.81 (0.17) | 44.55 (0.23) | 62.38 (0.43) | 64.39 (0.63) | 27.70 (0.23) | 28.34 (0.35) | 96.51 (0.08) | 96.51 (0.12) |
| SC | 49051 | 49.27 (0.25) | 52.68 (0.32) | 78.34 (0.18) | 59.91 (0.21) | 63.26 (0.39) | 66.19 (0.49) | 27.72 (0.23) | 30.14 (0.29) | 95.09 (0.09) | 95.65 (0.11) |
| ST | 52199 | 46.87 (0.36) | 53.01 (0.48) | 68.01 (0.29) | 55.92 (0.31) | 55.65 (0.58) | 60.99 (0.76) | 26.68 (0.32) | 31.16 (0.44) | 95.07 (0.13) | 95.76 (0.17) |
| OBC | 101786 | 48.99 (0.18) | 48.30 (0.25) | 79.78 (0.12) | 50.38 (0.15) | 61.68 (0.28) | 64.77 (0.38) | 26.96 (0.16) | 28.49 (0.22) | 95.46 (0.06) | 95.85 (0.08) |
| Don’t know | 11572 | 60.55 (0.53) | 64.08 (0.69) | 76.89 (0.40) | 54.95 (0.48) | 63.19 (0.89) | 66.45 (1.15) | 23.19 (0.46) | 26.63 (0.64) | 96.54 (0.18) | 96.95 (0.22) |
| ***Religion*** | | | | | | | | | | | |
| Hindus | 187573 | 51.62 (0.13) | 51.61 (0.18) | 80.79 (0.9) | 54.03 (0.11) | 62.53 (0.21) | 65.17 (0.28) | 27.08 (0.12) | 29.02 (0.16) | 95.51 (0.05) | 95.91 (0.06) |
| Muslims | 40950 | 49.82 (0.29) | 53.44 (0.43) | 69.21 (0.23) | 43.88 (0.24) | 55.17 (0.45) | 59.90 (0.67) | 25.37 (0.25) | 27.28 (0.38) | 95.61 (0.10) | 95.85 (0.15) |
| Christians | 20934 | 63.57 (0.77) | 67.20 (1.12) | 78.54 (0.57) | 43.21 (0.69) | 64.28 (1.29) | 68.47 (1.95) | 27.19 (0.71) | 30.54 (1.10) | 97.23 (0.23) | 96.89 (0.37) |
| Others | 10170 | 67.04 (0.64) | 67.87 (0.88) | 82.98 (0.45) | 52.55 (0.60) | 73.76 (1.04) | 75.01 (1.36) | 36.11 (0.65) | 38.22 (0.91) | 96.63 (0.22) | 96.96 (0.29) |
| ***Place of residence*** | | | | | | | | | | | |
| Rural | 198248 | 45.54 (0.14) | 47.83 (0.19) | 75.07 (0.10) | 54.38 (0.12) | 61.06 (0.22) | 64.87 (0.29) | 26.43 (0.12) | 28.99 (0.17) | 95.07 (0.05) | 95.65 (0.07) |
| Urban | 61379 | 67.41 (0.20) | 66.23 (0.29) | 88.68 (0.12) | 46.20 (0.19) | 62.99 (0.36) | 64.24 (0.51) | 28.59 (0.19) | 29.30 (0.29) | 96.92 (0.07) | 96.85 (0.09) |
| ***Region*** | | | | | | | | | | | |
| Northern | 48703 | 50.83 (0.32) | 52.17 (0.42) | 83.15 (0.21) | 59.04 (0.27) | 63.72 (0.51) | 67.22 (0.64) | 27.85 (0.29) | 28.50 (0.38) | 96.03 (0.11) | 96.16 (0.14) |
| Central | 75645 | 32.44 (0.22) | 32.44 (0.29) | 71.29 (0.17) | 51.78 (0.19) | 54.92 (0.36) | 57.71 (0.49) | 27.37 (0.20) | 28.06 (0.29) | 93.59 (0.09) | 94.32 (0.12) |
| Eastern | 54075 | 42.29 (0.23) | 45.91 (0.31) | 69.31 (0.18) | 52.89 (0.19) | 68.09 (0.35) | 72.81 (0.45) | 23.54 (0.19) | 27.58 (0.28) | 95.54 (0.08) | 96.24 (0.10) |
| North-eastern | 37167 | 50.24 (0.59) | 57.31 (0.76) | 67.99 (0.49) | 56.53 (0.53) | 52.26 (1.05) | 57.75 (1.38) | 22.86 (0.49) | 27.60 (0.69) | 95.55 (0.22) | 95.94 (0.28) |
| Western | 18276 | 72.48 (0.29) | 71.20 (0.44) | 89.73 (0.17) | 43.50 (0.28) | 54.21 (0.54) | 55.82 (0.79) | 27.32 (0.29) | 29.36 (0.45) | 97.07 (0.09) | 97.15 (0.14) |
| Southern | 25761 | 79.09 (0.29) | 78.77 (0.31) | 95.24 (0.10) | 51.51 (0.24) | 67.59 (0.42) | 67.99 (0.58) | 31.61 (0.25) | 33.30 (0.35) | 97.31 (0.08) | 97.09 (0.11) |
| *Note:* FHWE denotes Frontline Health Worker Interaction; SE denotes Standard Error; CFI denotes Child Full Immunization, and @ represents Public institutional delivery samples only. | | | | | | | | | | | |

| Table S4. Odds ratio by using Binary Logistic Regression (BLR) model of mother and child (0-5 years) health outcomes by levels of FHWE among the poor and non-poor women delivered in public health institutions in India, 2015-16 | | | | | | | | | | | | |
| --- | --- | --- | --- | --- | --- | --- | --- | --- | --- | --- | --- | --- |
| Predictor Variables | Antenatal Care (4 or > 4 times) | | | Place of delivery (Public Institutions) | | | Child Full Immunization (12-23 months) | | | Infant Postnatal care (within 2 days of delivery) | | |
|  | Odds Ratio (SE) | | | Odds Ratio (SE) | | | Odds Ratio (SE) | | | Odds Ratio (SE) | | |
|  | Poor  (*n*=51153) | Non-Poor (*n*=54462) | Total (*n*=105615) | Poor (*n*=130132) | Non-Poor (*n*=129495) | Total (*n*=259627) | Poor (*n*=20520) | Non-Poor (*n*=18783) | Total (*n*=39303) | Poor (*n*=51153) | Non-Poor (*n*=54462) | Total (*n*=105615) |
| **Panel A *(Unadjusted)*** | | | | | | | | | | | | |
| ***Level of FHWE*** | | | | | | | | | | | | |
| Low |  |  |  |  |  |  |  |  |  |  |  |  |
| Medium | 2.124^*^ (0.07) | 1.559^*^ (0.04) | 1.699^*^ (0.03) | 1.752^*^ (0.03) | 1.393^*^ (0.02) | 1.533^*^ (0.02) | 1.549^*^ (0.06) | 1.558^*^ (0.06) | 1.553^*^ (0.04) | 1.891^*^ (0.08) | 1.676^*^ (0.06) | 1.774^*^ (0.05) |
| High | 6.566^*^ (0.21) | 2.326^*^ (0.06) | 3.430^*^ (0.06) | 2.594^*^ (0.04) | 2.681^*^ (0.03) | 2.342^*^ (0.02) | 2.381^*^ (0.09) | 2.158^*^ (0.09) | 2.289^*^ (0.06) | 4.337^*^ (0.16) | 3.088^*^ (0.10) | 3.593^*^ (0.08) |
| **Panel B *(Adjusted)*** | | | | | | | | | | | | |
| ***Level of FHWE*** | | | | | | | | | | | | |
| Low |  |  |  |  |  |  |  |  |  |  |  |  |
| Medium | 1.976^*^ (0.07) | 1.560^*^ (0.05) | 1.685^*^ (0.04) | 1.655^*^ (0.02) | 1.368^*^ (0.02) | 1.494^*^ (0.02) | 1.539^*^ (0.06) | 1.544^*^ (0.07) | 1.550^*^ (0.04) | 1.881^*^ (0.08) | 1.677^*^ (0.06) | 1.766^*^ (0.05) |
| High | 5.560^*^ (0.19) | 2.357^*^ (0.07) | 3.485^*^ (0.07) | 2.408^*^ (0.04) | 2.079^*^ (0.03) | 2.226^*^ (0.02) | 2.325^*^ (0.09) | 2.128^*^ (0.09) | 2.255^*^ (0.07) | 4.312^*^ (0.16) | 3.087^*^ (0.09) | 3.608^*^ (0.09) |
| ***Birth Order*** | | |  | | |  | | |  | | |  |
| 1 |  |  |  |  |  |  |  |  |  |  |  |  |
| 2 | 0.789^*^(0.02) | 0.808^*^(0.02) | 0.794^*^ (0.01) | 0.780^*^(0.01) | 1.026^*^(0.01) | 0.925^*^ (0.01) | 0.858^*^(0.03) | 0.832^*^(0.03) | 0.840^*^ (0.02) | 1.037 (0.03) | 1.006 (0.02) | 1.013 (0.02) |
| 3 | 0.603^*^(0.02) | 0.642^*^(0.02) | 0.607^*^ (0.01) | 0.678^*^(0.01) | 1.011 (0.02) | 0.866^*^ (0.01) | 0.774^*^(0.04) | 0.612^*^(0.04) | 0.693^*^ (0.03) | 0.998 (0.04) | 1.016 (0.03) | 0.990 (0.02) |
| 3+ | 0.410^*^(0.02) | 0.469^*^(0.02) | 0.406^*^ (0.01) | 0.568^*^(0.01) | 0.989 (0.03) | 0.759^*^ (0.01) | 0.728^*^(0.04) | 0.526^*^(0.04) | 0.634^*^ (0.03) | 1.079^*^(0.04) | 0.999 (0.05) | 1.017 (0.03) |
| ***Current age of woman*** | | |  | | |  | | |  | | |  |
| 15-19 |  |  |  |  |  |  |  |  |  |  |  |  |
| 20-24 | 0.951 (0.05) | 1.073 (0.07) | 1.014 (0.04) | 0.990 (0.03) | 1.004 (0.04) | 0.956^*^ (0.02) | 1.165^*^(0.10) | 1.163 (0.12) | 1.176^*^ (0.08) | 1.163^*^ (0.06) | 0.993 (0.06) | 1.112^*^ (0.04) |
| 25-29 | 1.043 (0.06) | 1.262^*^ (0.08) | 1.188 (0.05) | 1.031 (0.04) | 0.909^*^(0.04) | 0.891^*^ (0.02) | 1.235^*^(0.11) | 1.426^*^ (0.16) | 1.346^*^ (0.09) | 1.194^*^ (0.07) | 1.002 (0.06) | 1.145^*^ (0.05) |
| 30-34 | 1.211^*^(0.07) | 1.577^*^ (0.10) | 1.457^*^ (0.06) | 1.079^*^(0.04) | 0.784^*^ (0.03) | 0.829^*^ (0.02) | 1.196^*^(0.11) | 1.361^*^ (0.16) | 1.302^*^ (0.09) | 1.234^*^ (0.08) | 1.219 (0.09) | 1.147^*^ (0.05) |
| 35-39 | 1.311^*^(0.09) | 1.526^*^ (0.12) | 1.485^*^ (0.07) | 0.943 (0.04) | 0.721^*^ (0.03) | 0.745^*^ (0.02) | 1.316^*^(0.15) | 1.959^*^ (0.29) | 1.586^*^ (0.14) | 1.279^*^ (0.09) | 1.219^*^ (0.09) | 1.313^*^ (0.07) |
| 40-44 | 1.264^*^(0.13) | 1.883^*^ (0.24) | 1.539^*^ (0.12) | 0.859^*^ (0.05) | 0.524^*^ (0.04) | 0.627^*^ (0.03) | 1.513^*^(0.26) | 2.027^*^(0.58) | 1.734^*^ (0.25) | 1.309^*^ (0.13) | 1.374^*^ (0.17) | 1.357^*^ (0.10) |
| 45-49 | 1.224 (0.19) | 1.186 (0.33) | 1.288^*^ (0.17) | 0.801^*^(0.06) | 0.417^*^ (0.06) | 0.567^*^ (0.04) | 1.216 (0.31) | 0.781 (0.66) | 1.282 (0.30) | 1.189 (0.18) | 1.361 (0.39) | 1.218 (0.16) |
| ***Education status of Women*** | | |  | | |  | | |  | | | |
| Illiterate |  |  |  |  |  |  |  |  |  |  |  |  |
| Primary | 1.459^*^(0.04) | 1.189^*^ (0.05) | 1.449^*^ (0.03) | 1.169^*^ (0.02) | 1.115^*^ (0.03) | 1.169^*^ (0.02) | 1.165^*^ (0.05) | 1.329^*^ (0.09) | 1.243^*^ (0.04) | 1.078^*^ (0.03) | 1.129^*^ (0.05) | 1.115^*^ (0.03) |
| Secondary | 1.601^*^ (0.04) | 1.553^*^ (0.05) | 1.835^*^ (0.04) | 1.460^*^ (0.02) | 1.044^*^(0.02) | 1.179^*^ (0.01) | 1.339^*^ (0.05) | 1.403^*^ (0.07) | 1.433^*^ (0.04) | 1.028 (0.03) | 1.219^*^ (0.04) | 1.170^*^ (0.02) |
| Higher | 1.496^*^ (0.12) | 1.789^*^ (0.08) | 2.187^*^ (0.07) | 1.192^*^ (0.06) | 0.530^*^(0.01) | 0.542^*^ (0.01) | 1.499^*^ (0.19) | 1.576^*^ (0.11) | 1.669^*^ (0.09) | 0.979 (0.08) | 1.305^*^ (0.06) | 1.282^*^ (0.02) |
| ***Education status of husband/ partner*** | | |  | | |  | | | | | | |
| Illiterate |  |  |  |  |  |  |  |  |  |  |  |  |
| Primary | 1.023 (0.08) | 1.140 (0.14) | 1.061 (0.03) | 1.138^*^ (0.05) | 1.054 (0.07) | 1.154^*^ (0.04) | 1.309^*^(0.15) | 1.122 (0.23) | 1.261^*^ (0.12) | 1.103 (0.08) | 0.909 (0.11) | 1.012 (0.06) |
| Secondary | 1.034 (0.07) | 1.236^*^(0.12) | 1.159^*^ (0.06) | 1.140^*^ (0.04) | 1.024 (0.06) | 1.092^*^ (0.03) | 1.231^*^(0.11) | 1.144 (0.18) | 1.256^*^ (0.09) | 1.057 (0.07) | 1.159 (0.12) | 1.102^*^ (0.06) |
| Higher | 1.139 (0.17) | 1.280^*^(0.15) | 1.287^*^ (0.09) | 1.111 (0.10) | 0.735^*^ (0.05) | 0.755^*^ (0.03) | 1.551^*^ (0.39) | 1.053 (0.20) | 1.290^*^ (0.16) | 0.949 (0.15) | 1.059 (0.13) | 1.018 (0.08) |
| Don't Know/missing | 0.952 (0.25) | 1.293 (0.41) | 1.103 (0.22) | 0.884 (0.14) | 1.059 (0.18) | 0.977 (0.11) | 1.434 (0.62) | 1.007 (0.47) | 1.271 (0.39) | 0.930 (0.26) | 0.621 (0.21) | 0.759 (0.16) |
| ***Occupational status of woman*** | | |  | | |  | | | | | | |
| Not working |  |  |  |  |  |  |  |  |  |  |  |  |
| White collar | 0.957 (0.24) | 0.539^*^ (0.07) | 0.609^*^ (0.07) | 1.129 (0.17) | 1.036 (0.07) | 1.045 (0.07) | 3.578^*^ (1.53) | 1.174 (0.33) | 1.674^*^ (0.39) | 1.352 (0.32) | 0.969 (0.13) | 1.025 (0.12) |
| Agricultural worker | 0.937 (0.07) | 0.963 (0.09) | 0.935 (0.05) | 0.854^*^ (0.03) | 0.909^*^(0.05) | 0.898^*^ (0.03) | 0.805^*^(0.08) | 0.853 (01.4) | 0.799^*^ (0.07) | 1.164^*^(0.08) | 1.167 (0.11) | 1.161^*^ (0.06) |
| Service/manual work | 1.393^*^(0.14) | 1.007 (0.10) | 1.191^*^ (0.09) | 0.881^*^(0.05) | 1.080 (0.06) | 0.984 (0.04) | 1.235 (0.19) | 1.053 (0.19) | 1.123 (0.16) | 1.466^*^ (0.14) | 1.206^*^(0.11) | 1.325^*^ (0.09) |
| Don’t know/missing | 0.835 (0.17) | 0.589^*^(0.15) | 0.712^*^ (0.11) | 1.066 (0.14) | 0.931 (0.13) | 1.020 (0.09) | 1.109 (0.39) | 0.787 (0.28) | 1.508 (0.19) | 1.483^*^(0.30) | 1.741^*^(0.43) | 1.585^*^ (0.25) |
| ***Occupational status of husband/ partner*** | | |  | | |  | | | | | | |
| Not working |  |  |  |  |  |  |  |  |  |  |  |  |
| White collar | 1.237 (0.19) | 1.065 (0.13) | 1.169^*^ (0.11) | 1.063 (0.09) | 0.641^*^ (0.05) | 0.756^*^ (0.04) | 0.951 (0.21) | 1.427^*^(0.26) | 1.202 (0.31) | 0.951 (0.14) | 1.051 (0.13) | 0.986 (0.09) |
| Agricultural worker | 1.259^*^(0.17) | 0.893 (0.11) | 1.063 (0.09) | 1.125 (0.08) | 0.807^*^ (0.06) | 0.931 (0.05) | 1.085 (0.21) | 1.357^*^(0.26) | 1.213 (0.16) | 0.774^*^ (0.10) | 0.968 (0.12) | 0.881 (0.08) |
| Service/manual work | 1.358^*^(0.19) | 1.248^*^(0.15) | 1.286^*^ (0.11) | 1.166^*^(0.09) | 0.737^*^ (0.05) | 0.886^*^ (0.04) | 1.278 (0.25) | 1.806^*^ (0.32) | 1.508^*^ (0.19) | 0.966 (0.13) | 1.337^*^ (0.01) | 1.153^*^ (0.10) |
| Don’t know/missing | 1.541^*^(0.39) | 1.465 (0.37) | 1.485^*^ (0.26) | 1.243 (0.17) | 0.767^*^(0.11) | 0.928 (0.09) | 0.783 (0.29) | 1.768 (0.72) | 1.156 (0.31) | 0.702 (0.19) | 1.219 (0.32) | 0.941 (0.17) |
| ***Caste*** | | | | | | | | | | | | |
| Others |  |  | |  |  |  |  |  |  |  |  |  |
| SC | 0.803^*^ (0.03) | 0.879^*^ (0.03) | 0.802^*^ (0.02) | 1.161^*^ (0.03) | 1.712^*^ (0.03) | 1.536^*^ (0.02) | 0.998 (0.06) | 1.065 (0.06) | 1.013 (0.04) | 1.071^*^(0.04) | 1.052 (0.13) | 1.019 (0.02) |
| ST | 0.943 (0.04) | 0.898^*^(0.04) | 0.907^*^ (0.03) | 0.988 (0.02) | 1.513^*^ (0.04) | 1.301^*^ (0.02) | 0.840 (0.05) | 0.967 (0.08) | 0.852^*^ (0.04) | 1.200^*^ (0.05) | 0.955 (0.04) | 1.067^*^ (0.03) |
| OBC | 0.635^*^ (0.02) | 0.801^*^ (0.02) | 0.698^*^ (0.01) | 1.068^*^ (0.02) | 1.122^*^ (0.02) | 1.157^*^ (0.01) | 1.001 (0.06) | 1.082^*^(0.05) | 1.030 (0.04) | 1.012 (0.04) | 1.031 (0.03) | 0.995 (0.02) |
| Don’t know/missing | 1.278^*^ (0.07) | 1.114^*^ (0.06) | 1.175^*^ (0.04) | 1.092^*^ (0.04) | 1.647^*^ (0.05) | 1.413^*^ (0.03) | 0.967 (0.09) | 1.156^*^(0.10) | 1.047 (0.07) | 0.997 (0.06) | 0.867^*^ (0.04) | 0.915^*^ (0.04) |
| ***Religion*** | | | | | | | | | | | | |
| Hindus |  |  |  |  |  |  |  |  |  |  |  |  |
| Muslims | 1.109^*^ (0.04) | 1.151^*^ (0.04) | 1.145^*^ (0.03) | 0.630^*^ (0.01) | 0.846^*^ (0.01) | 0.752^*^ (0.01) | 0.889^*^(0.05) | 0.906^*^(0.04) | 0.898^*^ (0.03) | 1.044 (0.04) | 1.104^*^ (0.03) | 1.078^*^ (0.03) |
| Christians | 1.086 (0.10) | 0.949 (0.07) | 1.038 (0.06) | 0.457^*^ (0.02) | 0.770^*^ (0.03) | 0.633^*^ (0.02) | 1.180 (0.19) | 1.261^*^(0.15) | 1.264^*^ (0.12) | 0.876 (0.08) | 1.079 (0.07) | 0.994 (0.05) |
| Others | 1.394^*^ (0.11) | 1.278^*^ (0.07) | 1.322^*^ (0.06) | 0.648^*^ (0.03) | 0.865^*^ (0.03) | 0.763^*^ (0.02) | 1.32^**^(0.17) | 1.579^*^ (0.16) | 1.523^*^ (0.12) | 1.099 (0.09) | 1.517^*^ (0.08) | 1.403^*^ (0.06) |
| ***Place of residence*** | | | | | | | | | | | | |
| Rural |  |  |  |  |  | |  |  |  |  |  |  |
| Urban | 1.615^*^ (0.06) | 1.428^*^ (0.03) | 1.741^*^ (0.03) | 1.288^*^ (0.03) | 0.927^*^ (0.01) | 0.913^*^ (0.01) | 1.097^*^(0.06) | 0.898^*^ (0.03) | 1.008 (0.03) | 1.016 (0.04) | 1.024 (0.02) | 1.092^*^ (0.02) |
| ***Region*** | | | | | | | | | | | | |
| Northern |  |  |  |  |  | |  |  |  |  |  |  |
| Central | 0.547^*^ (0.02) | 0.567^*^ (0.02) | 0.501^*^ (0.01) | 0.654^*^ (0.02) | 0.685^*^ (0.01) | 0.719^*^ (0.01) | 0.850^*^ (0.05) | 0.672^*^ (0.03) | 0.693^*^ (0.03) | 1.023 (0.04) | 1.056^*^(0.03) | 0.982 (0.02) |
| Eastern | 0.863^*^ (0.04) | 1.059^*^(0.03) | 0.796^*^ (0.02) | 0.579^*^ (0.01) | 0.718^*^ (0.01) | 0.681^*^ (0.01) | 1.684^*^ (0.10) | 1.318^*^(0.08) | 1.348^*^ (0.05) | 0.918^*^(0.04) | 1.045^*^(0.04) | 0.889^*^ (0.02) |
| North-eastern | 1.069 (0.06) | 1.265^*^ (0.08) | 0.992 (0.04) | 0.694^*^ (0.03) | 1.101^*^(0.05) | 0.866^*^ (0.02) | 0.746^*^ (0.07) | 0.544^*^ (0.06) | 0.585^*^ (0.04) | 1.111^*^(0.07) | 0.749^*^ (0.05) | 0.876^*^ (0.04) |
| Western | 2.347^*^ (0.13) | 1.921^*^ (0.07) | 1.921^*^ (0.06) | 0.485^*^ (0.02) | 0.452^*^ (0.01) | 0.469^*^ (0.01) | 0.747^*^ (0.06) | 0.493^*^ (0.03) | 0.559^*^ (0.03) | 1.079 (0.06) | 0.918^*^(0.03) | 0.931^*^ (0.03) |
| Southern | 3.444^*^ (0.19) | 2.575^*^ (0.08) | 2.667^*^ (0.07) | 0.731^*^ (0.02) | 0.594^*^ (0.01) | 0.602^*^ (0.01) | 0.994 (0.08) | 0.720^*^ (0.04) | 0.794^*^ (0.03) | 1.039 (0.05) | 1.022 (0.03) | 1.002 (0.03) |
| *Note:* SE represents Standard Error in the parentheses; ® Reference group; ^*^ *p < 0.05;* FHWE: Frontline Health Worker Interaction; CFI: Child Full Immunization. | | | | | | | | | | | | |

| Table S5. Hazard ratio by using Cox Proportional Hazard regression model of mother and child (0-5 years) health outcomes by FHWE Level, among the poor and non-poor women delivered in public health institutions in India, 2015-16 | | | |
| --- | --- | --- | --- |
| Predictor Variables | Hazard Ratio (SE) | | |
|  | Poor (*n*=71446) | Non-Poor (*n*=69582) | Total (*n*=141028) |
| **Panel A *(Unadjusted)*** | | | |
| ***Level of FHWE*** | | | |
| Low |  |  |  |
| Medium | 0.545^*^ (0.03) | 0.646^*^ (0.05) | 0.580^*^ (0.03) |
| High | 0.208^*^ (0.02) | 0.264^*^ (0.03) | 0.227^*^ (0.01) |
| **Panel B *(Adjusted)*** | | | |
| ***Level of FHWE*** | | | |
| Low |  |  |  |
| Medium | 0.547^*^ (0.04) | 0.625^*^ (0.5) | 0.576^*^ (0.03) |
| High | 0.210^*^ (0.02) | 0.248^*^ (0.2) | 0.225^*^ (0.01) |
| ***Birth Order*** | | | |
| 1 |  |  |  |
| 2 | 0.974 (0.78) | 1.221^*^(0.11) | 1.084 (0.07) |
| 3 | 1.149 (0.11) | 1.514^*^ (0.18) | 1.318^*^ (0.09) |
| 3+ | 1.261^*^(0.13) | 2.287^*^ (0.29) | 1.599^*^ (0.13) |
| ***Current age of woman*** | | | |
| 15-19 |  |  |  |
| 20-24 | 0.499^*^ (0.07) | 0.790 (0.22) | 0.542^*^ (0.07) |
| 25-29 | 0.452^*^ (0.07) | 0.612^*^(0.17) | 0.452^*^ (0.06) |
| 30-34 | 0.404^*^ (0.07) | 0.552^*^(0.16) | 0.402^*^ (0.06) |
| 35-39 | 0.427^*^ (0.08) | 0.581^*^(0.18) | 0.422^*^ (0.06) |
| 40-44 | 0.544^*^ (0.11) | 0.591 (0.22) | 0.501^*^ (0.09) |
| 45-49 | 0.559^*^ (0.18) | 0.554 (0.32) | 0.506^*^ (0.14) |
| ***Education status of Women*** | | | |
| Illiterate |  |  |  |
| Primary | 1.084 (0.08) | 0.964 (0.12) | 1.038 (0.07) |
| Secondary | 0.838^*^(0.06) | 0.779^*^(0.08) | 0.780^*^ (0.04) |
| Higher | 0.475^*^(0.16) | 0.562^*^ (0.09) | 0.503^*^ (0.07) |
| ***Education status of husband/ partner*** | | | |
| Illiterate |  |  |  |
| Primary | 0.998 (0.21) | 1.073 (0.34) | 1.087 (0.18) |
| Secondary | 0.988 (0.17) | 0.655 (0.18) | 0.910 (0.13) |
| Higher | 0.513 (0.31) | 0.398^*^(0.15) | 0.542^*^(0.15) |
| Don't Know/missing | 0.783 (0.54) | 0.479 (0.40) | 0.648 (0.35) |
| ***Occupational status of woman*** | | | |
| Not working |  |  |  |
| White collar | 1.003 (0.59) | 1.966^*^(0.67) | 1.587 (0.46) |
| Agricultural worker | 0.922 (0.18) | 1.139 (0.33) | 0.946 (0.15) |
| Service/manual work | 1.197 (0.30) | 1.831^*^(0.48) | 1.427^*^ (0.26) |
| Don’t know/missing | 0.793 (0.49) | 0.565 (0.44) | 0.756 (0.38) |
| ***Occupational status of husband/ partner*** | | | |
| Not working |  |  |  |
| White collar | 0.519^*^(0.02) | 1.605 (0.77) | 0.858 (0.24) |
| Agricultural worker | 0.616 (0.19) | 1.601 (0.77) | 0.860 (0.22) |
| Service/manual work | 0.659 (0.20) | 1.302 (0.61) | 0.838 (0.21) |
| Don’t know/missing | 1.060 (0.55) | 3.661^*^(2.39) | 1.666 (0.67) |
| ***Caste*** | | | |
| Others |  |  |  |
| SC | 1.144 (0.13) | 1.327^*^ (0.16) | 1.277^*^ (0.10) |
| ST | 1.274^*^(0.15) | 1.561^*^(0.24) | 1.442^*^ (0.13) |
| OBC | 0.932 (0.10) | 1.188 (0.13) | 1.067 (0.08) |
| Don’t know/missing | 1.022 (0.17) | 1.139 (0.22) | 1.092 (0.14) |
| ***Religion*** | | | |
| Hindus |  |  |  |
| Muslims | 1.059 (0.09) | 0.783^*^(0.09) | 0.953 (0.07) |
| Christians | 1.213 (0.19) | 1.496^*^(0.26) | 1.343^*^ (0.15) |
| Others | 0.831 (0.18) | 0.866 (0.16) | 0.834 (0.12) |
| ***Place of residence*** | | | |
| Rural |  |  |  |
| Urban | 1.059 (0.11) | 0.812^*^ (0.06) | 0.833^*^ (0.05) |
| ***Region*** | | | |
| Northern |  |  |  |
| Central | 1.390^*^(0.13) | 1.482^*^(0.14) | 1.472^*^ (0.09) |
| Eastern | 0.870 (0.09) | 0.804 (0.12) | 0.905 (0.07) |
| North-eastern | 0.868 (0.12) | 0.887 (0.14) | 0.942 (0.09) |
| Western | 0.746 (0.14) | 0.734 (0.14) | 0.758^*^ (0.10) |
| Southern | 1.109 (0.18) | 0.964 (0.13) | 1.028 (0.10) |
| *Note:* SE represents Standard Error in the parentheses; ® Reference group; ^*^ *p < 0.05;* FHWE: Frontline Health Worker Interaction. | | | |

| Table S6. Odds ratio by using Binary Logistic Regression (BLR) model of mother and child (0-5 years) health outcomes by levels of FHWE among the poor and non-poor **of rural women** in India, 2015-16 | | | | | | | | | | | | |
| --- | --- | --- | --- | --- | --- | --- | --- | --- | --- | --- | --- | --- |
| Predictor Variables | Antenatal Care (4 or > 4 times) | | | Place of delivery (Institutional) | | | Child Full Immunization (12-23 months) | | | Infant Postnatal care (within 2 days of delivery) | | |
|  | Odds Ratio (SE) | | | Odds Ratio (SE) | | | Odds Ratio (SE) | | | Odds Ratio (SE) | | |
|  | Poor  (*n*=83918) | Non-Poor (*n*=59147) | Total (*n*=143065) | Poor (*n*=120700) | Non-Poor (*n*=77548) | Total (*n*=198248) | Poor (*n*=34385) | Non-Poor (*n*=20989) | Total (*n*=55374) | Poor (*n*=83918) | Non-Poor (*n*=59147) | Total (*n*=143065) |
| **Panel A *(Unadjusted)*** | | | | | | | | | | | | |
| ***Level of FHWE*** | | | | | | | | | | | | |
| Low |  |  |  |  |  |  |  |  |  |  |  |  |
| Medium | 2.524^*^ (0.07) | 1.815^*^ (0.05) | 2.254^*^ (0.04) | 1.807^*^ (0.03) | 1.706^*^ (0.05) | 1.902^*^ (0.03) | 1.706^*^ (0.05) | 1.549^*^ (0.06) | 1.708^*^ (0.04) | 2.003^*^ (0.06) | 1.732^*^ (0.06) | 1.962^*^ (0.04) |
| High | 8.735^*^ (0.22) | 3.369^*^ (0.08) | 5.846^*^ (0.09) | 2.843^*^ (0.05) | 3.049^*^ (0.09) | 3.231^*^ (0.04) | 2.802^*^ (0.08) | 2.158^*^ (0.08) | 2.678^*^ (0.06) | 4.837^*^ (0.13) | 3.193^*^ (0.09) | 4.221^*^ (0.09) |
| **Panel B *(Adjusted)*** | | | | | | | | | | | | |
| ***Level of FHWE*** | | | | | | | | | | | | |
| Low |  |  |  |  |  |  |  |  |  |  |  |  |
| Medium | 2.234^*^ (0.06) | 1.685^*^ (0.05) | 1.945^*^ (0.04) | 1.688^*^ (0.03) | 1.461^*^ (0.04) | 1.644^*^ (0.02) | 1.663^*^ (0.05) | 1.599^*^ (0.07) | 1.659^*^ (0.04) | 1.978^*^ (0.06) | 1.735^*^ (0.06) | 1.899^*^ (0.04) |
| High | 6.682^*^ (0.18) | 2.816^*^ (0.08) | 4.496^*^ (0.08) | 2.583^*^ (0.05) | 2.477^*^ (0.08) | 2.597^*^ (0.04) | 2.664^*^ (0.08) | 2.204^*^ (0.09) | 2.527^*^ (0.06) | 4.817^*^ (0.14) | 3.288^*^ (0.10) | 4.089^*^ (0.09) |
| ***Birth Order*** | | |  | | |  | | |  | | |  |
| 1 |  |  |  |  |  |  |  |  |  |  |  |  |
| 2 | 0.740^*^(0.02) | 0.762^*^(0.02) | 0.741^*^ (0.01) | 0.584^*^(0.01) | 0.508^*^(0.02) | 0.557^*^ (0.01) | 0.830^*^(0.03) | 0.878^*^(0.03) | 0.841^*^ (0.02) | 0.971 (0.02) | 0.958^*^ (0.02) | 0.957 (0.02) |
| 3 | 0.578^*^(0.02) | 0.524^*^(0.02) | 0.535^*^ (0.01) | 0.467^*^(0.01) | 0.358 (0.01) | 0.424^*^ (0.01) | 0.784^*^(0.03) | 0.724^*^(0.04) | 0.751^*^ (0.02) | 0.925^*^ (0.03) | 0.901^*^ (0.03) | 0.895 (0.02) |
| 3+ | 0.428^*^(0.14) | 0.394^*^(0.02) | 0.377^*^ (0.01) | 0.387^*^(0.01) | 0.295 (0.01) | 0.344^*^ (0.01) | 0.729^*^(0.04) | 0.679^*^(0.05) | 0.686^*^ (0.03) | 0.954 (0.03) | 0.859 (0.04) | 0.873 (0.02) |
| ***Current age of woman*** | | |  | | |  | | |  | | |  |
| 15-19 |  |  |  |  |  |  |  |  |  |  |  |  |
| 20-24 | 0.968 (0.04) | 1.100 (0.06) | 1.047 (0.04) | 0.988 (0.04) | 0.857 (0.08) | 0.995 (0.04) | 1.126 (0.08) | 0.889 (0.09) | 1.058 (0.06) | 1.109^*^ (0.05) | 1.069 (0.06) | 1.116^*^ (0.04) |
| 25-29 | 1.079 (0.05) | 1.384^*^(0.08) | 1.284 (0.05) | 1.054 (0.05) | 1.031 (0.04) | 1.126^*^ (0.04) | 1.202^*^(0.08) | 1.038 (0.11) | 1.182^*^ (0.07) | 1.143^*^ (0.06) | 1.156^*^ (0.07) | 1.200^*^ (0.04) |
| 30-34 | 1.182^*^(0.06) | 1.659^*^(0.10) | 1.499^*^ (0.06) | 1.141^*^(0.05) | 1.339^*^ (0.13) | 1.293^*^ (0.05) | 1.198^*^(0.09) | 1.044^*^ (0.12) | 1.193^*^ (0.07) | 1.117^*^ (0.06) | 1.149^*^ (0.07) | 1.195^*^ (0.05) |
| 35-39 | 1.245^*^(0.07) | 1.895^*^(0.14) | 1.603^*^ (0.07) | 1.037 (0.05) | 1.403^*^ (0.15) | 1.198^*^ (0.05) | 1.124 (0.09) | 1.252^*^ (0.17) | 1.193^*^ (0.08) | 1.171^*^ (0.07) | 1.353^*^ (0.09) | 1.308^*^ (0.06) |
| 40-44 | 1.105 (0.09) | 1.943^*^(0.22) | 1.462^*^ (0.09) | 0.901 (0.06) | 1.057 (0.15) | 1.015 (0.03) | 1.292^*^(0.15) | 1.378^*^(0.34) | 1.319^*^ (0.14) | 1.166^*^ (0.09) | 1.397^*^ (0.16) | 1.290^*^ (0.08) |
| 45-49 | 0.919 (0.11) | 1.305 (0.29) | 1.116 (0.12) | 0.782^*^(0.06) | 0.835 (0.18) | 0.865^*^ (0.07) | 1.389 (0.24) | 1.449 (0.86) | 1.374 (0.22) | 0.924 (0.11) | 1.752 (0.37) | 1.097 (0.11) |
| ***Education status of Women*** | | |  | | |  | | |  | | | |
| Illiterate |  |  |  |  |  |  |  |  |  |  |  |  |
| Primary | 1.559^*^(0.04) | 1.309^*^(0.05) | 1.589^*^ (0.03) | 1.122^*^ (0.02) | 1.125^*^ (0.04) | 1.236^*^ (0.02) | 1.262^*^ (0.04) | 1.233^*^ (0.08) | 1.312^*^ (0.04) | 1.074^*^ (0.03) | 1.138^*^ (0.05) | 1.125^*^ (0.02) |
| Secondary | 1.771^*^ (0.04) | 1.677^*^(0.05) | 2.089^*^ (0.04) | 1.805^*^ (0.03) | 1.901^*^(0.06) | 2.124^*^ (0.03) | 1.454^*^ (0.04) | 1.357^*^ (0.07) | 1.579^*^ (0.04) | 1.048^*^ (0.02) | 1.198^*^ (0.04) | 1.194^*^ (0.02) |
| Higher | 2.155^*^ (0.15) | 2.044^*^(0.08) | 2.924^*^ (0.09) | 3.182^*^ (0.25) | 3.501^*^(0.21) | 4.574^*^ (0.07) | 1.610^*^ (0.17) | 1.457^*^ (0.09) | 1.811^*^ (0.08) | 1.106 (0.08) | 1.291^*^ (0.05) | 1.371^*^ (0.02) |
| ***Education status of husband/ partner*** | | |  | | |  | | | | | | |
| Illiterate |  |  |  |  |  |  |  |  |  |  |  |  |
| Primary | 1.063 (0.08) | 1.044 (0.13) | 1.125^*^ (0.06) | 1.117^*^ (0.05) | 0.834 (0.11) | 1.063 (0.05) | 1.091 (0.09) | 1.329 (0.24) | 1.102 (0.08) | 1.049 (0.07) | 0.929 (0.11) | 1.025 (0.02) |
| Secondary | 1.117^*^(0.06) | 0.991 (0.10) | 1.201^*^ (0.05) | 1.238^*^ (0.05) | 1.166 (0.13) | 1.272^*^ (0.05) | 1.224^*^(0.08) | 1.797^*^ (0.25) | 1.332^*^ (0.08) | 1.045 (0.06) | 1.111 (0.11) | 1.114^*^(0.05) |
| Higher | 1.068 (0.13) | 0.078 (0.12) | 1.394^*^ (0.09) | 1.652 (0.19) | 1.382^*^ (0.19) | 1.697^*^ (0.13) | 1.183 (0.21) | 1.474^*^ (0.24) | 1.181^*^ (0.11) | 1.081 (0.15) | 1.019 (0.13) | 1.071 (0.07) |
| Don't Know/missing | 1.279 (0.31) | 0.914 (0.28) | 1.205 (0.22) | 1.358 (0.25) | 2.215^*^ (0.81) | 1.619 (0.26) | 1.049 (0.36) | 1.739 (0.78) | 1.219 (0.33) | 1.088 (0.26) | 0.893 (0.27) | 1.037 (0.19) |
| ***Occupational status of woman*** | | |  | | |  | | | | | | |
| Not working |  |  |  |  |  |  |  |  |  |  |  |  |
| White collar | 0.714 (0.15) | 0.798 (0.10) | 0.774^*^ (0.08) | 1.104 (0.19) | 1.007 (0.21) | 1.081 (0.15) | 3.348^*^ (1.23) | 1.181 (0.30) | 1.743^*^ (0.37) | 1.326 (0.27) | 1.339^*^ (0.15) | 1.319 (0.13) |
| Agricultural worker | 0.941 (0.05) | 0.985 (0.08) | 0.933 (0.04) | 0.853^*^ (0.04) | 0.729^*^(0.07) | 0.810^*^ (0.03) | 0.941 (0.07) | 0.932 (0.12) | 0.919^*^ (0.06) | 1.124^*^(0.06) | 1.215^*^ (0.09) | 1.140^*^(0.05) |
| Service/manual work | 1.162 (0.09) | 1.281^*^(0.14) | 1.196^*^ (0.08) | 0.860^*^ (0.06) | 0.774^*^(0.09) | 0.845 (0.05) | 1.225 (0.16) | 0.857 (0.16) | 1.090 (0.11) | 1.260^*^ (0.11) | 1.187^*^(0.11) | 1.225^*^(0.08) |
| Don’t know/missing | 1..022 (0.19) | 0.591^*^(0.15) | 0.799 (0.12) | 0.805 (0.12) | 0.506^*^ (0.16) | 0.695 (0.09) | 1.023 (0.29) | 0.949 (0.31) | 0.975 (0.21) | 1.027 (0.19) | 1.424 (0.34) | 1.163 (0.17) |
| ***Occupational status of husband/ partner*** | | |  | | |  | | | | | | |
| Not working |  |  |  |  |  |  |  |  |  |  |  |  |
| White collar | 1.016 (0.13) | 1.469^*^(0.17) | 1.319^*^ (0.11) | 1.087 (0.11) | 0.799 (0.14) | 1.078 (0.09) | 0.822 (0.14) | 0.983 (0.19) | 0.909 (0.12) | 1.181 (0.15) | 1.175 (0.14) | 1.188 (0.10) |
| Agricultural worker | 1.047 (0.12) | 1.141 (0.13) | 1.086 (0.09) | 0.968 (0.08) | 0.716^*^ (0.13) | 0.888 (0.07) | 0.939 (0.14) | 1.041 (0.26) | 0.957 (0.11) | 0.974 (0.11) | 1.091 (0.13) | 1.023 (0.09) |
| Service/manual work | 1.134 (0.13) | 1.573^*^(0.18) | 1.286^*^ (0.11) | 1.141 (0.09) | 0.697^*^ (0.12) | 1.028 (0.04) | 1.132 (0.17) | 1.161 (0.22) | 1.132 (0.13) | 1.243 (0.14) | 1.407^*^(0.17) | 1.329^*^(0.11) |
| Don’t know/missing | 0.853^*^(0.19) | 2.159 (0.53) | 1.321^*^ (0.21) | 1.027 (0.16) | 0.680^*^(0.21) | 0.942 (0.13) | 0.998 (0.28) | 0.974 (0.36) | 0.966 (0.22) | 1.069 (0.24) | 1.040 (0.25) | 1.067 (0.18) |
| ***Caste*** | | | | | | | | | | | | |
| Others |  |  | |  |  |  |  |  |  |  |  |  |
| SC | 0.744^*^(0.02) | 0.791^*^ (0.02) | 0.706^*^ (0.02) | 0.966 (0.03) | 0.731^*^ (0.03) | 0.819^*^ (0.02) | 0.986 (0.05) | 1.116 (0.06) | 0.988 (0.03) | 1.072^*^(0.04) | 1.007 (0.03) | 0.970 (0.02) |
| ST | 0.829^*^ (0.03) | 0.839^*^ (0.04) | 0.772^*^ (0.02) | 0.618 (0.02) | 0.482^*^ (0.03) | 0.508^*^ (0.01) | 0.779^*^ (0.04) | 0.951 (0.07) | 0.764^*^ (0.03) | 1.083^*^(0.04) | 0.846^*^ (0.04) | 0.934^*^(0.02) |
| OBC | 0.643^*^ (0.02) | 0.758^*^ (0.02) | 0.667^*^ (0.01) | 1.063^*^ (0.03) | 0.868^*^ (0.03) | 0.945^*^ (0.02) | 0.972 (0.04) | 1.065 (0.04) | 0.988 (0.03) | 1.083 (0.03) | 0.946^*^ (0.02) | 0.971 (0.02) |
| Don’t know/missing | 1.100^*^ (0.05) | 1.226^*^(0.07) | 1.088^*^ (0.04) | 0.869^*^ (0.03) | 0.979 (0.07) | 0.828^*^ (0.03) | 1.066 (0.08) | 1.109 (0.10) | 1.059 (0.06) | 0.885 (0.05) | 0.873^*^(0.05) | 0.848^*^ (0.03) |
| ***Religion*** | | | | | | | | | | | | |
| Hindus |  |  |  |  |  |  |  |  |  |  |  |  |
| Muslims | 1.079^*^ (0.03) | 1.251^*^ (0.04) | 1.172^*^ (0.03) | 0.559^*^ (0.01) | 0.621^*^ (0.02) | 0.579^*^ (0.01) | 0.797^*^ (0.03) | 0.871^*^(0.04) | 0.831^*^ (0.02) | 0.968 (0.03) | 1.219^*^ (0.04) | 1.079^*^ (0.02) |
| Christians | 1.039 (0.07) | 0.818 (0.06) | 0.982 (0.05) | 0.578^*^ (0.03) | 0.774^*^ (0.07) | 0.694^*^ (0.02) | 1.218 (0.13) | 1.218^*^(0.14) | 1.269^*^ (0.09) | 0.870 (0.06) | 1.056 (0.07) | 1.002 (0.05) |
| Others | 1.431^*^ (0.09) | 1.556^*^ (0.08) | 1.498^*^ (0.06) | 0.563^*^ (0.03) | 1.279^*^ (0.09) | 0.751^*^ (0.03) | 1.439^*^ (0.13) | 2.126^*^ (0.22) | 1.795^*^ (0.12) | 1.152 (0.07) | 1.579^*^ (0.08) | 1.423^*^ (0.05) |
| ***Region*** | | | | | | | | | | | | |
| Northern |  |  |  |  |  | |  |  |  |  |  |  |
| Central | 0.556^*^ (0.02) | 0.697^*^ (0.02) | 0.564^*^ (0.01) | 0.536^*^ (0.02) | 0.661^*^ (0.02) | 0.533^*^ (0.01) | 0.924 (0.04) | 0.842^*^ (0.04) | 0.798^*^ (0.03) | 1.056 (0.04) | 1.245^*^(0.04) | 1.058^*^ (0.02) |
| Eastern | 0.832^*^ (0.03) | 1.157^*^(0.04) | 0.808^*^ (0.02) | 0.472^*^ (0.01) | 0.756^*^ (0.03) | 0.463^*^ (0.01) | 1.734^*^ (0.08) | 1.693^*^(0.10) | 1.449^*^ (0.05) | 0.855^*^(0.03) | 0.955 (0.03) | 0.805^*^ (0.02) |
| North-eastern | 0.937 (0.05) | 1.330^*^ (0.08) | 0.906^*^ (0.03) | 0.445^*^ (0.02) | 0.719^*^(0.05) | 0.433^*^ (0.01) | 0.718^*^ (0.05) | 0.584^*^ (0.06) | 0.587^*^ (0.03) | 1.000^*^(0.05) | 0.778^*^ (0.05) | 0.810^*^ (0.03) |
| Western | 2.179^*^ (0.09) | 2.526^*^ (0.09) | 2.236^*^ (0.06) | 0.881^*^ (0.03) | 1.677^*^ (0.08) | 1.072^*^ (0.03) | 0.647^*^ (0.04) | 0.569^*^ (0.03) | 0.574^*^ (0.02) | 1.115 (0.05) | 0.889^*^(0.03) | 0.924^*^ (0.03) |
| Southern | 3.531^*^ (0.16) | 3.479^*^ (0.11) | 3.421^*^ (0.09) | 1.362^*^ (0.06) | 2.901^*^ (0.14) | 1.913^*^ (0.06) | 1.131 (0.07) | 0.841^*^ (0.04) | 0.921^*^ (0.04) | 0.962 (0.04) | 1.039 (0.03) | 0.988 (0.03) |
| *Note:* SE represents Standard Error in the parentheses; ® Reference group; ^*^ *p < 0.05;* FHWE: Frontline Health Worker Interaction; CFI: Child Full Immunization. | | | | | | | | | | | | |

| Table S7. Hazard ratio by using Cox Proportional Hazard regression model of child survival outcome by FHWE Level, among the poor and non-poor **of rural women** in India, 2015-16 | | | |
| --- | --- | --- | --- |
| Predictor Variables | Hazard Ratio (SE) | | |
|  | Poor (*n*=120700) | Non-Poor (*n*=77548) | Total (*n*=198248) |
| **Panel A *(Unadjusted)*** | | | |
| ***Level of FHWE*** | | | |
| Low |  |  |  |
| Medium | 0.582^*^  (0.02) | 0.580^*^ (0.02) | 0.571^*^  (0.01) |
| High | 0.333^*^  (0.01) | 0.290^*^ (0.02) | 0.305^*^  (0.01) |
| **Panel B *(Adjusted)*** | | | |
| ***Level of FHWE*** | | | |
| Low |  |  |  |
| Medium | 0.556^*^  (0.02) | 0.578^*^ (0.02) | 0.565^*^  (0.01) |
| High | 0.321^*^  (0.01) | 0.297^*^ (0.02) | 0.314^*^  (0.01) |
| ***Birth Order*** | | | |
| 1 |  |  |  |
| 2 | 0.989 (0.03) | 1.256^*^ (0.06) | 1.079^*^ (0.03) |
| 3 | 1.258^*^ (0.05) | 1.595^*^ (0.10) | 1.374^*^ (0.05) |
| 3+ | 1.778^*^ (0.08) | 2.653^*^ (0.20) | 2.002^*^ (0.08) |
| ***Current age of woman*** | | | |
| 15-19 |  |  |  |
| 20-24 | 0.320^*^ (0.02) | 0.248^*^ (0.03) | 0.296^*^ (0.02) |
| 25-29 | 0.172^*^ (0.01) | 0.130^*^ (0.01) | 0.156^*^ (0.01) |
| 30-34 | 0.133^*^ (0.01) | 0.098^*^ (0.01) | 0.119^*^ (0.01) |
| 35-39 | 0.131^*^ (0.01) | 0.092^*^ (0.01) | 0.115^*^ (0.01) |
| 40-44 | 0.137^*^ (0.01) | 0.130^*^ (0.02) | 0.128^*^ (0.01) |
| 45-49 | 0.167^*^ (0.02) | 0.153^*^ (0.04) | 0.153^*^ (0.02) |
| ***Education status of Women*** | | | |
| Illiterate |  |  |  |
| Primary | 1.072^*^ (0.04) | 0.967 (0.06) | 1.052 (0.03) |
| Secondary | 1.078^*^ (0.03) | 0.907^*^ (0.05) | 0.989 (0.03) |
| Higher | 1.179 (0.14) | 0.966 (0.08) | 0.969 (0.06) |
| ***Education status of husband/ partner*** | | | |
| Illiterate |  |  |  |
| Primary | 0.839^*^ (0.07) | 1.384 (0.27) | 0.939 (0.07) |
| Secondary | 0.824^*^ (0.06) | 0.839 (0.14) | 0.823^*^ (0.05) |
| Higher | 0.622^*^ (0.13) | 0.757 (0.15) | 0.695^*^ (0.08) |
| Don't Know/missing | 0.516^*^ (0.16) | 0.955 (0.55) | 0.584^*^ (0.16) |
| ***Occupational status of woman*** | | | |
| Not working |  |  |  |
| White collar | 1.131 (0.29) | 0.802 (0.23) | 0.962 (0.18) |
| Agricultural worker | 0.949 (0.07) | 1,142 (0.16) | 0.982 (0.07) |
| Service/manual work | 0.914 (0.11) | 1.137 (0.21) | 0.966 (0.09) |
| Don’t know/missing | 1.222 (0.33) | 1.369 (0.60) | 1.241 (0.28) |
| ***Occupational status of husband/ partner*** | | | |
| Not working |  |  |  |
| White collar | 0.867 (0.16) | 0.599^*^ (0.12) | 0.776^*^ (0.10) |
| Agricultural worker | 0.958 (0.15) | 0.628^*^ (0.12) | 0.835^*^ (0.10) |
| Service/manual work | 1.025 (0.16) | 0.552^*^ (0.11) | 0.847^*^ (0.10) |
| Don’t know/missing | 1.316 (0.33) | 0.408 (0.21) | 0.989^*^ (0.22) |
| ***Caste*** | | | |
| Others |  |  |  |
| SC | 1.145^*^ (0.06) | 1.290^*^ (0.08) | 1.229 (0.05) |
| ST | 1.160^*^ (0.06) | 1.197^*^ (0.10) | 1.214 (0.05) |
| OBC | 1.028 (0.05) | 1.063 (0.06) | 1.063 (0.04) |
| Don’t know/missing | 0.966 (0.07) | 1.104 (0.12) | 1.034 (0.06) |
| ***Religion*** | | | |
| Hindus |  |  |  |
| Muslims | 0.960 (0.04) | 0.894 (0.05) | 0.958^*^ (0.03) |
| Christians | 0.617^*^ (0.04) | 0.955 (0.11) | 0.699^*^ (0.04) |
| Others | 0.695^*^ (0.06) | 1.10 (0.10) | 0.839^*^ (0.05) |
| ***Region*** | | | |
| Northern |  |  |  |
| Central | 1.356^*^ (0.06) | 1.529^*^ (0.08) | 1.436 (0.05) |
| Eastern | 1.122^*^ (0.05) | 1.020 (0.07) | 1.150 (0.04) |
| North-eastern | 1.004 (0.06) | 0.835 (0.08) | 0.995^*^ (0.05) |
| Western | 0.876 (0.07) | 0.903 (0.08) | 0.894^*^ (0.05) |
| Southern | 1.093 (0.09) | 0.863^*^ (0.06) | 0.927^*^ (0.05) |
| *Note:* SE represents Standard Error in the parentheses; ® Reference group; ^*^ *p < 0.05;* FHWE: Frontline Health Worker Interaction. | | | |
